# Division-induced DNA double strand breaks in the chromosome terminus region of *Escherichia coli* lacking RecBCD DNA repair enzyme

**DOI:** 10.1371/journal.pgen.1006895

**Published:** 2017-10-02

**Authors:** Anurag Kumar Sinha, Adeline Durand, Jean-Michel Desfontaines, Ielyzaveta Iurchenko, Hélène Auger, David R. F. Leach, François-Xavier Barre, Bénédicte Michel

**Affiliations:** 1 Bacterial DNA stability, Genome biology department, Institute for Integrative Biology of the Cell (I2BC), CEA, CNRS, Université Paris-Sud, Université Paris-Saclay, Gif-sur-Yvette, France; 2 Evolution and maintenance of circular chromosomes, Genome biology department, Institute for Integrative Biology of the Cell (I2BC), CEA, CNRS, Université Paris-Sud, Université Paris-Saclay, Gif-sur-Yvette, France; 3 Institute of Cell Biology, School of Biological Sciences, University of Edinburgh, Edinburgh, United Kingdom; 4 High-throughput Sequencing facility, Institute for Integrative Biology of the Cell (I2BC), CEA, CNRS, Université Paris-Sud, Université Paris-Saclay, Gif-sur-Yvette, France; University of Washington School of Medicine, UNITED STATES

## Abstract

Marker frequency analysis of the *Escherichia coli recB* mutant chromosome has revealed a deficit of DNA in a specific zone of the terminus, centred on the *dif/TerC* region. Using fluorescence microscopy of a marked chromosomal site, we show that the *dif* region is lost after replication completion, at the time of cell division, in one daughter cell only, and that the phenomenon is transmitted to progeny. Analysis by marker frequency and microscopy shows that the position of DNA loss is not defined by the replication fork merging point since it still occurs in the *dif/TerC* region when the replication fork trap is displaced in strains harbouring ectopic *Ter* sites. Terminus DNA loss in the *recB* mutant is also independent of dimer resolution by XerCD at *dif* and of Topo IV action close to *dif*. It occurs in the terminus region, at the point of inversion of the GC skew, which is also the point of convergence of specific sequence motifs like KOPS and Chi sites, regardless of whether the convergence of GC skew is at *dif* (wild-type) or a newly created sequence. In the absence of FtsK-driven DNA translocation, terminus DNA loss is less precisely targeted to the KOPS convergence sequence, but occurs at a similar frequency and follows the same pattern as in FtsK^+^ cells. Importantly, using *ftsIts*, *ftsAts* division mutants and cephalexin treated cells, we show that DNA loss of the *dif* region in the *recB* mutant is decreased by the inactivation of cell division. We propose that it results from septum-induced chromosome breakage, and largely contributes to the low viability of the *recB* mutant.

## Author summary

RecBCD protein complex is an important player of DSB repair in bacteria and bacteria that cannot repair DNA double-stranded breaks (DSB) have a low viability. Whole genome sequencing analyses showed a deficit in specific sequences of the chromosome terminus region in *recB* mutant cells, suggesting terminus DNA degradation during growth. We studied here the phenomenon of terminus DNA loss by whole genome sequencing and microscopy analyses of exponentially growing bacteria. We tested all processes known to take place in the chromosome terminus region for a putative role in DNA loss: replication fork termination, dimer resolution, resolution of catenated chromosomes, and translocation of the chromosome arms in daughter cells during septum formation. None of the mutations that affect these processes prevents the phenomenon. However, we observed that terminus DNA loss is abolished in cells that cannot divide. We propose that in cells defective for RecBCD-mediated DSB repair the terminus region of the chromosome remains in the way of the growing septum during cell division, then septum closure triggers chromosome breakage and, in turn, DNA degradation.

## Introduction

**Fig 1:**
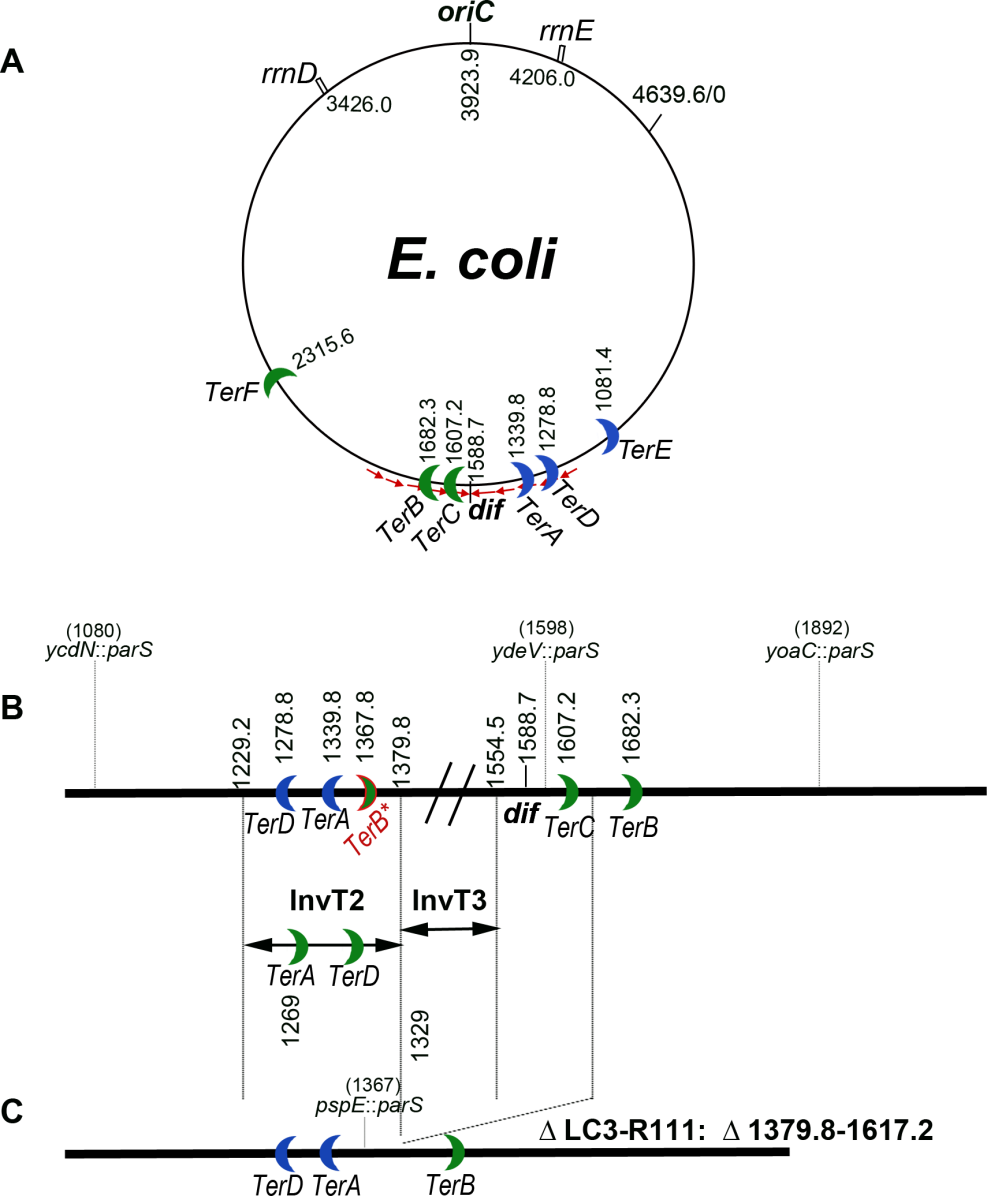
Schematic representation of the *E*. *coli* chromosome. A: circular map of the *E*. *coli* chromosome, *oriC*, *dif*, *rrnD* and *rrnE*, and *TerE* to *TerF* sites are indicated. The small red arrows show the orientation of KOPS sequences, which converge at *dif*. Numbers refer to the chromosome coordinates (in kb) of MG1655. B: linear map of the terminus region. Chromosome coordinates are increasing from left to right, as in the marker frequency panels shown in Figs 2, 3, 5 and 7, therefore in the opposite direction to the circular map. In addition to *dif* and *Ter* sites, the positions of the *parS*_pMT1_ sites used for microscopy experiments and the InvT2 and InvT3 endpoints are indicated. C: linear map of the ΔLC3-R111 deleted strain. Maps are not drawn to scale.

Most bacteria have a circular chromosome on which replication is initiated at single origin *oriC* and proceeds bi-directionally on the two replichores until forks meet in the terminus, opposite to *oriC*. The chromosome terminus is a particularly active region where several important processes take place: replication termination, chromosome dimer resolution and last steps of chromosome segregation (Fig 1A). In *E*. *coli*, replication is arrested by the presence of sites called *Ter* that are bound by a specific protein Tus (reviewed in [1, 2]). *Ter*-Tus complexes allow replication to proceed in one direction only and thus create a replication fork trap in which replication forks enter but from which they infrequently exit. This system allows right and left replichores to be replicated principally in a clockwise and anti-clockwise direction, respectively, ensuring that replication is mainly co-directional with transcription [3–6]. 2D-gel analyses allowed the visualisation of replication forks arrested at *TerC*, and to a lesser extent at *TerA* and *TerB* [7].

In most bacterial species a site called *dif*, which is located opposite to *oriC* on the circular chromosome, is acted upon by the XerC/XerD site-specific recombination complex to resolve chromosome dimers (reviewed in [8, 9]). *dif* is also the site of inversion of the GC skew on the chromosome [2, 10] and the site of orientation inversion of biologically active motifs such as Chi (crossover hotspot instigator) and KOPS (FtsK-orienting polar sequences [11, 12] reviewed in [13]). KOPS (GGGNAGGG) are used by the septum protein FtsK to orient the translocation of chromosomes to daughter cells, and the convergence of these sequences at *dif* makes it the last segregated chromosomal sequence in slow growing cells [14]. Although KOPS are present all around the chromosome, FtsK is particularly active in the 400 kb region, centred on *dif* [15]. Translocation of chromosomes by FtsK is arrested by encounter with the XerCD-*dif* complex [16, 17]; FtsK then activates this complex to trigger chromosome dimer resolution at *dif* [18–20]. Finally, FtsK was proposed to displace the terminus-specific DNA-bound protein MatP [14], a protein that organizes and condenses the 780 kb Ter macrodomain by binding specifically to short DNA sequences called *matS* [21–23]. In this manuscript we call “terminus” the chromosome region opposite to *oriC*, centred on the point of inversion of the GC skew, regardless of the position of replication forks merging.

The septum forms at mid-cell by the assembly of several proteins in a defined order (reviewed in [24, 25]). Early proteins are FtsZ and its regulators, which include FtsA. Formation of the Z-ring is essential for the recruitment of late proteins, including FtsK and FtsI. FtsK is a bi-functional protein, its N-terminal domain is essential for cell division and is anchored in the septum; it is separated by a linker from the C-terminal domain, a cytoplasmic ATPase non-essential for viability and responsible for (i) DNA translocation in a direction imposed by KOPS, and (ii) activation of chromosome dimer resolution by interaction with XerCD (reviewed in [26, 27]). FtsI is a septum peptidase essential for constriction (reviewed in [28, 29]).

The terminus region was reported to be a preferential region of genetic instability [30–33]. However, hyper-recombination in the terminus region was dependent on replication termination at Ter sites, or perturbation of the dimer resolution system XerCD/*dif*, or perturbation of FtsK-mediated chromosome segregation, and it occurred in a small subpopulation of cells (at most 1%) [30–33]. More recently, a limited region of the terminus was reported to be amplified in certain mutant contexts (notably in a *recG* mutant) and a nearly identical region was lost in *recB* mutants [34, 35].

RecBCD is the enzyme that initiates recombinational repair of DNA double-strand breaks (DSB) in *E*. *coli*. This enzyme specifically recognizes DNA double-strand ends, unwinds and cleaves dsDNA *via* its coupled DNA helicase and exonuclease (exo V) activities, and when it encounters a Chi site it loads RecA on single-stranded DNA (ssDNA) [36–38]. Loss of terminus DNA in *recB* mutants was proposed to result from the formation of dsDNA ends by erroneous merging of replication forks leading to a transient over-replicated intermediate [35]. According to the proposed model, in the RecBCD^+^ context over-replicated dsDNA ends would be appropriately degraded by RecBCD, restoring intact chromosomes, while in the *recB* mutant extensive DNA degradation from these dsDNA ends by various single-stranded DNA exonucleases would cause DNA loss [35].

The amplification of terminal DNA in a *recG* mutant has been examined in detail [6, 34, 39, 40], while the DNA loss in the *recB* mutant has been less extensively explored, and the model of merging forks has not been directly tested experimentally [35, 41]. Here we use Marker Frequency Analysis (MFA) and live-cell fluorescence microscopy to further characterize this phenomenon. As previously proposed [34, 35], we consider that loss of terminus DNA in the *recB* mutant results from the degradation of unrepaired DNA double-stranded ends, but we show that it is independent of the position of replication termination, which argues against the model of merging forks. In search for an alternative source of chromosome breakage, we show that terminus DNA loss in the *recB* mutant occurs during cell division and requires septum formation. We propose that chromosome breakage in the *E*. *coli* terminus region is septum-induced damage. In addition, we observed weak Tus-dependent DNA loss at *Ter* sites, which was only detected when division was prevented by mutation or when replication terminates at an ectopic *Ter*.

## Results

### Terminus DNA loss occurs in a sub-population of *recB* cells during cell division and is hereditary

**Fig 2:**
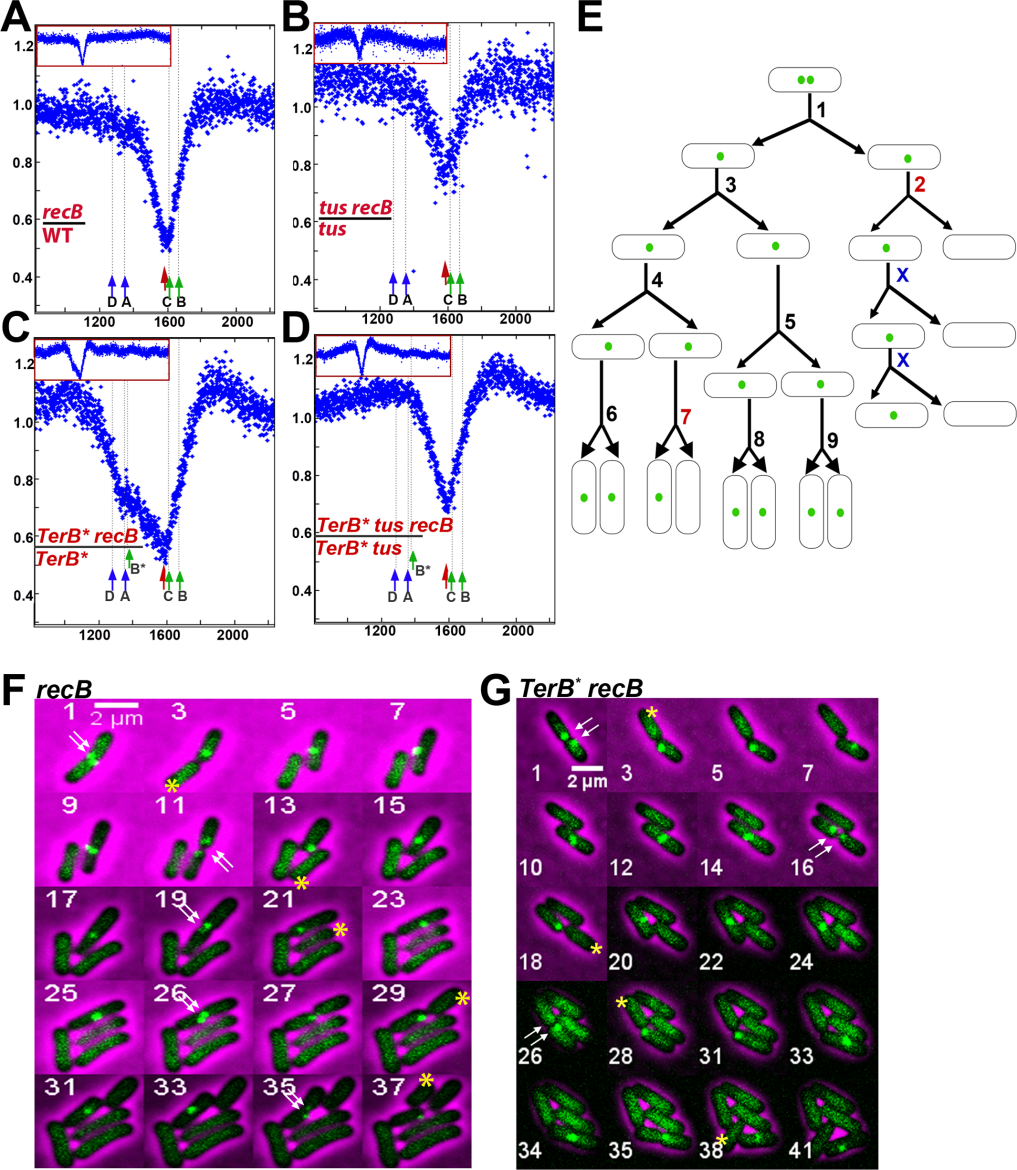
Loss of terminus DNA in the *recB* mutant is independent of the site of replication termination. A-D Sequence read frequencies of exponential phase cells normalized to the total number of reads were calculated for each strain. Ratios of normalized reads in isogenic RecB^+^ and *recB* mutants are plotted against chromosomal coordinates (in kb). Profile ratios of the terminus region are enlarged and profiles of the corresponding entire chromosomes are shown in insets. Original normalized profiles used to calculate ratios are shown in S1 and S2 Figs. The position of *dif* is indicated by a red arrow. *Ter* sites that arrest clockwise forks (*TerC*, *TerB*, TerB*, green arrow) and counter-clockwise forks (*TerA*, *TerD*, blue arrow) are shown. E. Schematic representation of the counting of initial events. Divisions producing two focus-containing cells and first divisions producing one focus-containing cell and one focus-less cell were counted; the ratio of the latter to the total number of divisions corresponds to the percentage of initial events shown in Table 1. In the scheme shown here, we have counted two initial events (divisions # 2 and 7) out of a total of 9 divisions (“X” are divisions of inherited focus loss and were not counted). Division #2 is clearly hereditary, while division # 7 is counted as an initial event but is not taken into account to calculate the ratio of hereditary events among initial events, since the progeny cannot be seen. F. Micrographs showing an example of focus loss during growth of a *recB* mutant. Time-lapse experiments were carried out on M9 glucose agarose pads at 30°C with pictures taken every 10 min. All cells contain *ydeV*::*parS*_pMT1_ and express the ParB_pMT1_ protein from the gene inserted into the chromosome. In this example a cell that generates a focus-less cell at the first generation has been cropped. The numbers in the upper left corner of the pictures indicate the frame number in the video. For reasons of space limitations only one out of two frames is shown (odd numbered frames are shown only), with the exception of frame 26. Other examples of focus-less cell production from a cropped bacterium, but for which all frames taken every 10 min are shown, can be seen in S1 and S2 Videos. The double white arrows indicate the presence of two foci before division, which shows that focus loss results from the degradation of a DNA sequence that has been previously replicated. The yellow stars show cells that have lost the focus following division. These focus-less cells generally do not divide while the sister cell that has kept the *ydeV*:: *parS*_pMT1_ site (the top cell in pictures 3 and 5 in this example) keeps growing and generates a focus-less cell at each division (pictures 3, 13, 21, 29, 37). G Micrographs showing an example of focus loss during growth of a TerB* *recB* mutant. Frame numbers are at the bottom left of each frame. Arrows and stars are as in F. A focus-less cell is produced at each generation, at frames 3, 18, 28, and 38.

Marker frequency analysis (MFA) of the chromosome of wild-type and *recB* mutant cells in exponential growth confirmed a deficit of DNA reads in the terminus region of the chromosome in the absence of RecB (Fig 2A, S1 Fig). To allow a direct comparison of MFA and microscopy results, all experiments were done in minimal medium (M9). This DNA loss is centred on the *dif-TerC* region when cells are grown exponentially in M9 glucose (Fig 2A), as previously reported for cells grown in LB [34, 35]. We have developed a live-cell microscopy approach to confirm that this phenomenon, which is observed by MFA in a population of growing cells, in fact occurs in a sub-population of individual cells and we have quantified this sub-population. Strains constitutively expressing the yGFP-ParB_pMT1_ protein from a chromosomal-borne gene and carrying *parS*_pMT1_ sites inserted at three different locations were used. Binding of the yGFP-ParB protein to its *parS* target site allows the visualisation of each chromosome *parS* sequence as a fluorescent focus. Three strain backgrounds carrying each a different *parS* sites were used. These *parS* sites were *ydeV*::parS_pMT1_ located between *dif* and *TerC*, 10 kb away from each site, *yoaC*::*parS*_pMT1_ located about 300 kb away from *dif* on the left replichore and *ycdN*::*parS*_pMT1_ located about 500 kb away from *dif* on the right replichore ([42], S1 Table, Fig 1B). Cells grown in exponential phase in M9 glucose medium were observed by fluorescence microscopy. In a wild-type context, nearly all cells showed foci and the proportion of cells with two foci increased with the distance from *dif*, as previously reported (S2 Table) [14, 43]. In the *recB* mutant, the proportion of cells without any focus was much higher than in RecB^+^ cells: the *recB* mutant with *ydeV*::*parS*_pMT1_ (the locus between *dif* and *TerC*) showed 32% of cells with no focus and the control *parS* sites located 300 or 500 kb from *dif* showed 7–8% of cells with no focus (Table 1, S2 Table). These results argue that in a *recB* context about one third of cells have lost the *dif-TerC* region specifically, in agreement with the results of MFA experiments [34, 35] (Fig 2A).

**Table 1:** The sequence close to *dif* is specifically lost in *recB* mutants.

Genotype	% cells with 0 focus		initial events	transmitted*
	*ydeV*::*parS*_pMT1_	*yoaC*::*parS*_pMT1_	*ydeV*::*parS*_pMT1_	
wild-type	0.6 ± 0.2	0.6 ± 0.3		
*recB*	32 ± 1.5	7.9 ± 1	17.7% (350)	74.5%
*recC*	30.6 ± 1.6	7.8 ± 1.6		
*recD*	0.63 ± 0.74	0.34 ± 0.37		
*tus*	0.8 ± 0.24	2.5 ± 0.3		
*tus recB*	36.7 ± 2.1	13.1 ± 0.8	15.8% (240)	87.1%
*pspE*::*TerB*	0.3 ± 0.3			
*pspE*::*TerB recB*	48 ± 7	8.8 ± 7	25.3% (383)	80.65%
*pspE*::*TerB tus*	0.8 ± 0.06			
*pspE*::*TerB tus recB*	35.1 ± 5.6			
*xerC*	14.8 ± 1	1.7 ± 1.4		
*xerC recB*	40.4 ± 0.9	14 ± 2.6		
*dif*	15.2 ± 1.8			
*dif recB*	41.5 ± 2		17.5% (498)	83.9%
*dif hipA*	15.7 ± 2.5	3.6		
*dif hipA recB*	64.6 ± 7	40 ± 3.2		
*ftsK*^*ATPase*^	20.6 ± 0.4	5.9 ± 0.6		
*ftsK*^*ATPase*^ *recB*	54.7 ± 0.1	14 ± 0.08		
*ftsKΔCter*	25.1 ± 1.9	4.5 ± 2.3		
*ftsKΔCter recB*	54.4 ± 1.2	15.9 ± 3.1	15.8% (303)	82.8%
*ftsK*^*ATPase*^ *tus*	27.3 ± 0.7			
*ftsK*^*ATPase*^ *tus recB*	49.8 ± 1.2			
*recB* [pET28]	34.2 ± 2.4			
*recB* [pET-parC-TD]	32.4 ± 4.7			
*endA recB*^a^	35.2 ± 3.7			
InvT3	4.9 ± 2.1			
InvT3 *recB*	38.4 ± 3.6			
InvT2	8.7 ± 3.7			
InvT2 *recB*	43.5 ± 3.4			

(a) experiment in the *endA recB* mutant was realized with the ParB protein expressed from plasmid pFHC2973 [42].

**Fig 3:**
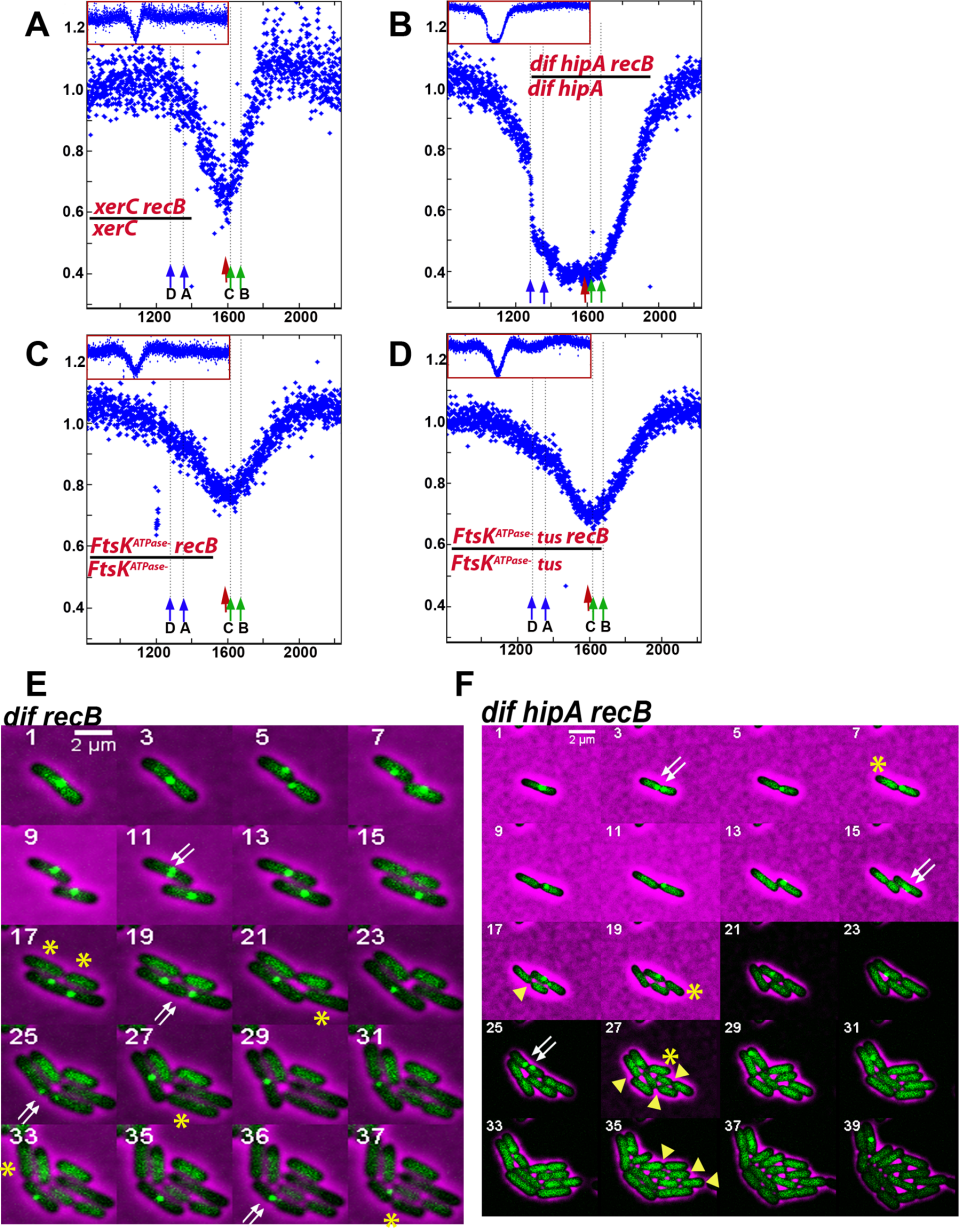
Loss of terminus DNA in the *recB* mutant is independent dimer resolution and FtsK translocation activity. A-D Sequence read frequencies of exponential phase cells normalized to the total number of reads were calculated for each strain. Ratios of normalized reads in isogenic RecB^+^ and *recB* mutants are plotted against chromosomal coordinates (in kb). Profile ratios of the terminus region are enlarged and profiles of the corresponding entire chromosomes are shown in insets. Original normalized profiles used to calculate ratios are shown in S3 Fig. The positions of *dif*, or of inversion of the GC skew at the new junction in *dif* mutants, is indicated by a red arrow. *Ter* sites that arrest clockwise forks (*TerC*, *TerB*, green arrow) and counter-clockwise forks (*TerA*, *TerD*, blue arrow) are shown. E-F. Time lapse experiments were carried out as described in the legend of Fig 2 and in the Material and Method section. As in Fig 2, the double white arrow indicate the two foci prior to foci loss, and the single yellow start indicates the focus-less cell formed in an hereditary way after cell division. In the *dif recB* mutant an example where both daughter cells lose the *ydeV*:: *parS*_pMT1_ site at the time of division is also shown with a double yellow star (dimer guillotining, frame 17). In the *dif hipA recB* mutant focus-less cells keep growing and dividing, which is indicated by yellow arrowheads.

Examples of time lapse experiments are shown in Figs 2, 3 and 6 and in S1–S7 Videos. For analysis, the number of divisions providing two foci-containing cells and the number of first divisions providing one focus-containing and one focus-less cells were manually counted (see Fig 2E and Material and Methods). Initial events shows the percentage of divisions that lead to one cell with a focus and one cell without focus, not counting the ones that follow a first event. The total number of counted divisions is between parentheses. Results are the sum of two experiments.

(*) The transmission to progeny was calculated on the events for which at least one additional division could be observed (50–80% of the initial events). Only fully hereditary events are counted, about 10–15% were hereditary but with an interruption of transmission for one generation and are not included here. Few (5–10%) occurred once with no evidence for transmission.

To better characterize this chromosomal DNA loss, the dynamic behaviour of foci was tracked by time lapse microscopy of *recB* cells growing on M9 glucose agarose pads, as described in Materials and Methods. As shown in Fig 2F and S1 and S2 Videos, *ydeV*::*parS*_pMT1_ foci were lost with the following characteristics: 1) the foci disappeared concomitantly with cell division, most often at the site of septum formation, and in one of the two daughter cells only (yellow stars), no loss at any other time point was observed, 2) the loss occurred after duplication of this region, since most of the time two foci were clearly visible at earlier time points (white arrows), 3) after cell division, the daughter cell that had lost a focus stopped growing and did not divide, whereas the cell that retained a focus divided again, and produced a focus-less cell at each generation after the first event (yellow stars). Although the production of focus-less cells was asymmetrical and hereditary, we did not observe any bias toward the old or new pole. We called the first division that produces one focus-containing cell and one focus-free cell “the initial event” and calculated that these represented 17.7% of cell divisions (not counting the “secondary events” that follow, Table 1). Because divisions that produced focus-less cells also produced a focus-containing cell, the proportion of focus-less cells in the population was expected to be one half of the proportion of divisions that produced them in the absence of transmission of the phenomenon to progeny. In contrast to this, the proportion of focus-less cells (32%) was higher than the proportion of divisions that produced them (17.7%), in agreement with the transmission of the phenomenon to following generations (Table 1). Nearly 75% of the initial events were transmitted to the progeny for as many generations as we could see (up to five). In addition, in about 17% of the cases transmission was interrupted for one generation (*i*.*e*. the cell that retained a focus produced two focus-containing cells, one propagated normally and the other one resumed the production of one focus-less cell at each generation). Although there is clearly some transmission in spite of the interruption, this second category of events was observed in all *recB* mutants and is not counted as transmitted in Table 1.

RecBCD has two activities, a recombinase activity that requires RecB and RecC but not RecD (helicase and RecA loading activities), and an exonuclease activity, called exoV, which degrades linear dsDNA and is catalyzed by the entire RecBCD complex [36, 38]. DNA degradation by exoV occurs in the absence of RecA or in the absence of Chi sites. *recD* mutant cells are recombination proficient but do not degrade dsDNA, and it was previously reported that terminus DNA loss is not detected by MFA in the *recD* mutant [35]. Accordingly, analysis of *ydeV-parS*_pMT1_ foci showed that the *recD* single mutant behaved like wild-type (Table 1), therefore we confirm that the presence of RecBC prevents DNA loss regardless of exoV activity. In contrast, the *recC* mutant that lacks both RecBC and RecBCD complexes and thus both recombination and exoV activities, behaved as the *recB* mutant (Table 1, S1 Fig).

### DNA loss in the *dif-TerC* region is independent of the replication arrest protein Tus, and of the position of replication forks merging

DNA loss was previously shown by MFA to occur in the *dif-TerC* region of *recB* mutants in two different *E*. *coli* genetic backgrounds: MG1655 [34], in which replication terminates primarily at *TerC* and 4 to 5 times less often at *TerA* [7], and in W3110 [35]. W3110 carries a large inversion between *rrnD* and *rrnE* around the replication origin, which enlarges the right replichore and shortens the left one by about 220 kb [44, 45] (Fig 1A). As a consequence of this inversion, the closest replication terminator from *oriC* in this context is *TerA* and not *TerC*, and therefore replication is expected to terminate at *TerA* more often than at *TerC*. Nonetheless, the position of the peak of DNA loss was the same in W3110 as previously reported in MG1655 [35]. This surprising observation prompted us to directly measure the influence of the position of replication termination on DNA loss in the *dif*/*TerC* region. For that purpose, we first compared *recB* and *recB tus* mutants (in which Ter sites are non-functional) by MFA (Fig 2B, S1 Fig) and by snap-shot fluorescence microscopy of *ydeV*::*parS*_pMT1_/yGFP-ParB_pMT1_ foci (Table 1). Inactivation of *tus* did not prevent DNA loss in the *dif-TerC* region detected by MFA (Fig 2B) and did not modify the percentage of focus-less cells for the *ydeV*::*parS*_pMT1_ locus close to *dif*, and for the *yoaC*::*parS*_pMT1_control locus (Table 1). Note that the ratio of reads in *tus recB* over the *tus* mutant increased in a large *Ter* region compared to the rest of the chromosome (Fig 2B). We do not know the reasons for this phenomenon, but the existence of a mixed population partially masks DNA loss at *dif/TerC* in the MFA experiment. Time lapse experiments showed that the loss of *ydeV*::*parS*_pMT1_ foci followed the same scheme in *tus recB* as previously observed in Tus^+^ *recB* cells, *i*.*e*. loss of focus in one daughter cell, at the septum, at the time of division, and transmission of this defect to the progeny (S3 Video), with a similar percentage of initial events (Table 1, 15.8% vs 17.7%), and a high level of events transmitted to progeny (87%). Replication was previously shown by 2D gels to terminate mainly at *TerC* in wild-type cells [7], but our result in the *tus* mutant suggests that most of the loss of DNA in the *dif-TerC* region occurs independently of forks merging at *TerC*.

Because in the *tus* mutant forks may still merge in the *dif* region that is opposite to the origin, we constructed a strain in which the clockwise replication forks are prevented from reaching *TerC* by the introduction of an additional *TerB* site that arrests replication prematurely, 29 kb downstream of *TerA* (*pspE*::*TerB*, TerB* in Fig 1A and 1B). In the TerB* strain a new replication fork trap is created between *TerA* and TerB* and the *dif* site is mainly replicated by the counter-clockwise fork, instead of the clockwise fork in the wild-type strain. Fluorescence microscopy showed that loss of *ydeV*::*parS*_pMT1_ foci (close to *dif*) was increased to 48% in the strain containing TerB*, while loss of control *yoaC*::*parS*_pMT1_ and *ydvN*::*parS*_pMT1_ loci was unchanged (Table 1, S2 Table). As expected, the increase from 32% in *recB* to 48% in TerB* recB cells was Tus-dependent, as we counted 35% of *ydeV*::*parS*_pMT1_ focus-less cells in the TerB* recB tus mutant (Table 1, S2 Table). Time lapse experiments showed that in TerB* recB cells *ydeV*::*parS*_pMT1_ foci were still lost in one daughter cell, at the septum, at the time of division, with a transmission of this defect to the progeny (Fig 2G, Table 1), and the proportion of original divisions that yielded the first focus-less cells in an inheritable manner was increased from 17.7% to 25% (Table 1). Finally, measuring loss of control *yoaC*::*parS*_pMT1_ foci (Table 1) and MFA analysis showed that the position of the peak of DNA loss at *dif* was not affected by the creation of this new replication fork trap, away from *TerC* (Fig 2C, S2 Fig). Nevertheless, the MFA experiment also revealed a new Tus-dependent peak of DNA loss at TerB*, weaker than the DNA loss at *dif* (Fig 2C and 2D, S2 Fig). It was previously proposed that DNA double-strand ends, target for RecBCD, were formed in the terminus region of the *recB* mutant by erroneous merging of replication forks, and that degradation of these unrepaired DNA double-stranded ends by the combined action of helicases and single-stranded exonucleases was the cause of terminus DNA loss [34, 35]. Since in the presence of the additional TerB* site, replication forks are expected to merge at this site or at *TerA*, and are therefore unlikely to merge at *TerC*, the strong DNA loss that we observed in the *dif/TerC* region of the TerB* *recB* mutant (Fig 2C, Table 1) cannot result from replication fork merging. We also propose that DNA loss results from DNA degradation by the combined action of helicases and single-stranded exonucleases, but propose that the dsDNA ends on which these enzymes act are produced by a DNA DSB occurring in *dif*/*TerC* region of the *recB* mutant chromosome, regardless of the position of replication termination. In addition, our results also show that displacing replication termination to TerB* creates a new hotspot of DNA loss, weaker than the *dif/TerC* hotspot, at the new replication termination site.

### DNA loss in the *dif/TerC* region is independent of dimer resolution

The observation that the position of DNA loss in the *dif-TerC* region is independent of the position of replication fork merging raises the possibility that it might be determined by *dif* rather than *TerC*. *dif* is the site of chromosome dimers resolution, a XerCD- and FtsK- dependent reaction [18–20]. We tested a possible role of dimer resolution in DNA loss by inactivating *xerC* or removing the *dif* site. In RecB^*+*^ cells these mutations increased the proportion of *ydeV*::*parS*_pMT1_ focus-less cells from less than 1% to about 15% (Table 1, S2 Table) and, accordingly, a weak DNA loss of the *dif* region could be detected by MFA (S3 Fig). Time lapse experiments showed that focus-loss in the *dif* or *xerC* single mutants results from breakage of both chromosomes at the time of cell division (Fig 3E, S4 Video); Abnormal pattern of cell division in microcolonies of *dif* and *xer* mutants was previously observed and was proposed to result from breakage of chromosome dimers by septum closure, which was called guillotining [46, 47]. 40–42% of cells lacked *ydeV*::*parS*_pMT1_ in *xerC recB* or *dif recB* mutants (Table 1). We propose that this higher level of focus-less cells compared to the *recB* single mutant results from a combination of broken dimers and septum-induced breaks (about 50% of dimers are RecB-independent, [48]). Accordingly, time lapse microscopy confirmed that some focus-less cells result from the concomitant loss of both *ydeV*::*parS*_pMT1_ foci in the two daughter cells at the time of division (presumably dimer breakage), whereas most of them result from the transmitted, asymmetric loss of one focus in one daughter cell at the time of division (Fig 3E). Interestingly, in *dif* or *xer* cells that contain a dimer, cell division was delayed for more than an hour before we observed cell separation and focus-loss (S4 Video), while cell division of the *recB* mutant was not delayed upon focus-loss in one daughter cell compared to cells that do not lose foci. The proportion of initial events in the *dif recB* mutant was 17.4%, similar to the *recB* single mutant, confirming that division-dependent loss of *ydeV*::*parS*_pMT1_ foci is independent from dimer resolution, and most of these events were transmitted to progeny (Table 1). Furthermore, the weaker loss of the control *yoaC*::*parS*_pMT1_ and *ycdN*::*parS*_pMT1_ foci compared to loss of the *dif* proximal *ydeV*::*parS*_pMT1_ site (Table 1, S2 Table), and MFA analyses of *xerC* and *xerC recB* mutant (Fig 3A, S3 Fig) confirmed that the loss of DNA remains centred on *dif* in the absence of dimer resolution.

Two *dif* deletions were tested: one that lacks only the *dif* site, and one that also inactivates the adjacent *hipA* locus. HipA is a toxin that blocks growth by inactivating translation and is counteracted by the short-lived anti-toxin HipB ([49] and ref therein). In the absence of both *dif* and HipA, the peak of DNA loss observed in MFA experiments in a *recB* mutant context was deeper and larger than in the *recB* single mutant (Fig 3B, S3 Fig). Accordingly, in microscopy snapshot experiments the proportion of cells that lack the *ydeV*::*parS*_pMT1_ or the control *yoaC*::*parS*_pMT1_ focus was much increased, to 65% and 40% respectively (Table 1, S2 Table). Time lapse experiments showed that in the absence of HipA, focus-less cells grew and divided for several generations, which increased the proportion of these cells in the population, and presumably allowed the degradation of more and more chromosomal DNA with generations (Fig 3F). This result showed that in *recB* single mutants the growth arrest of focus-less cells results from the degradation of the *hipAB* locus after chromosome breakage, which causes the accumulation of active toxin HipA. This phenomenon was previously described after breakage of chromosome dimers at the septum in a *dif* mutant [46]. It confirms genetically that chromosomes lacking the *ydeV*::*parS*_pMT1_ site originally conserve all genes required for growth and cell division, and that chromosome degradation is a slow process (essential genes are absent from the terminus region, [47, 50]).

### The region of terminus DNA loss is enlarged in cells deficient for the *ftsK* translocase activity

If DNA loss results from DSBs occurring at the peak of loss followed by nearly symmetrical DNA degradation by single-stranded nucleases, why are these DSBs introduced in the *dif* region even in the absence of this site? The *dif* site is the last chromosome locus segregated in daughter cells because it is the site of convergence of the KOPS sequences, which are used by the FtsK protein to segregate replicated chromosomes to daughter cells ([14, 27], Fig 1A). KOPS sites are present all around the chromosome but FtsK is mainly active in a 400 kb region approximating the one of decreased reads in the *recB* mutant [15]. We tested a putative role of FtsK in the localisation of terminus breaks with the use of a *ftsK*^*ATPase*^ mutant, in which *ftsK* carries a nucleotide substitution that specifically inactivates the ATPase activity and thus prevents DNA translocation without affecting DNA binding. We first analyzed *ftsK*^*ATPase*^ RecB^+^ cells by microscopy. Quantification of focus loss showed that the proportion of focus-less cells was increased compared to wild-type cells, particularly for the *ydeV*::parS_pMT1_ site located next to the *dif* locus (from 0.6% to about 20%, Table 1, S2 Table). This DNA loss presumably resulted mainly from a lack of dimer resolution in the absence of FtsK translocation activity. Inactivation of *recB* in the *ftsK*^*ATPase*^ mutant led to a large increase in focus-less cells (nearly 55% of cells contain no *ydeV-parS*_pMT1_ focus and 14% contain no *yoaC-parS*_pMT1_ focus). Similar results were observed when FtsK translocation was inactivated by the deletion the entire protein C-terminal domain (Table 1, S2 Table). Time lapse microscopy showed two kinds of events leading to focus loss in the *ftsK recB* mutant context. In 15.5% of the divisions, one *ydeV-parS*_pMT1_ focus was lost at the septum, at the division time, in one daughter cell only, with a transmission of this defect to the progeny (Table 1, S5 Video). This result shows that the events occurring in the *recB* single mutant also occur in *ftsK*^*ATPase*^ *recB* cells, with a similar frequency (Table 1). In addition, the two daughter cells lost the *ydeV-parS*_pMT1_ foci during 12% of the divisions, presumably owing to dimer breakage (S5 Video), and other types of focus loss could be observed, which presumably result from the segregation defect and account for the high percentage of focus-less cells in the *ftsK*^Δ*CTer*^ *recB* mutant population (S6 and S7 Videos).

In contrast with the *dif* and *xerC* mutants, DNA loss around *dif* was not detected by MFA in the *ftsK*^*ATPase*^ single mutant, and rather a weak DNA amplification was visible in the terminus region (S3 Fig). Since microscopy results show a loss of the *dif* region in 20% of *ftsK*^*ATPase*^ and *ftsK*^Δ*CTer*^ mutants, this amplification reflects the existence of a mixed population of cells, some that lose the *dif/TerC* region as observed by microscopy, and some that amplify it and mask the loss in the MFA experiments. DNA degradation around the *dif* locus was observed by MFA in the *ftsK*^*ATPase*^ *recB* mutant (Fig 3C, S3 Fig), but a larger DNA region was degraded than in the *recB* single mutant (compare Fig 3C and Fig 2A). Furthermore, the MFA did not show the deep loss expected from the microscopy results. This loss could be masked if DNA amplification occurs in a subset of cells, as detected by MFA in single *ftsK*^*ATPase*^ mutant. We conclude that the DNA translocation activity of FtsK plays an important role in the sharp targeting of DNA loss to the *dif* region in the *recB* mutant, leading to a wider distribution of DNA loss in the absence of the FtsK C-terminal domain or ATPase activity. Nevertheless, DNA loss and therefore DNA breaks still occur specifically in the *dif/TerC* chromosome region when DNA translocation by FtsK is inactivated.

### Absence of DNA double-strand breaks (DSBs) in the *dif/TerC* region from RecBCD^+^ cells

**Fig 4:**
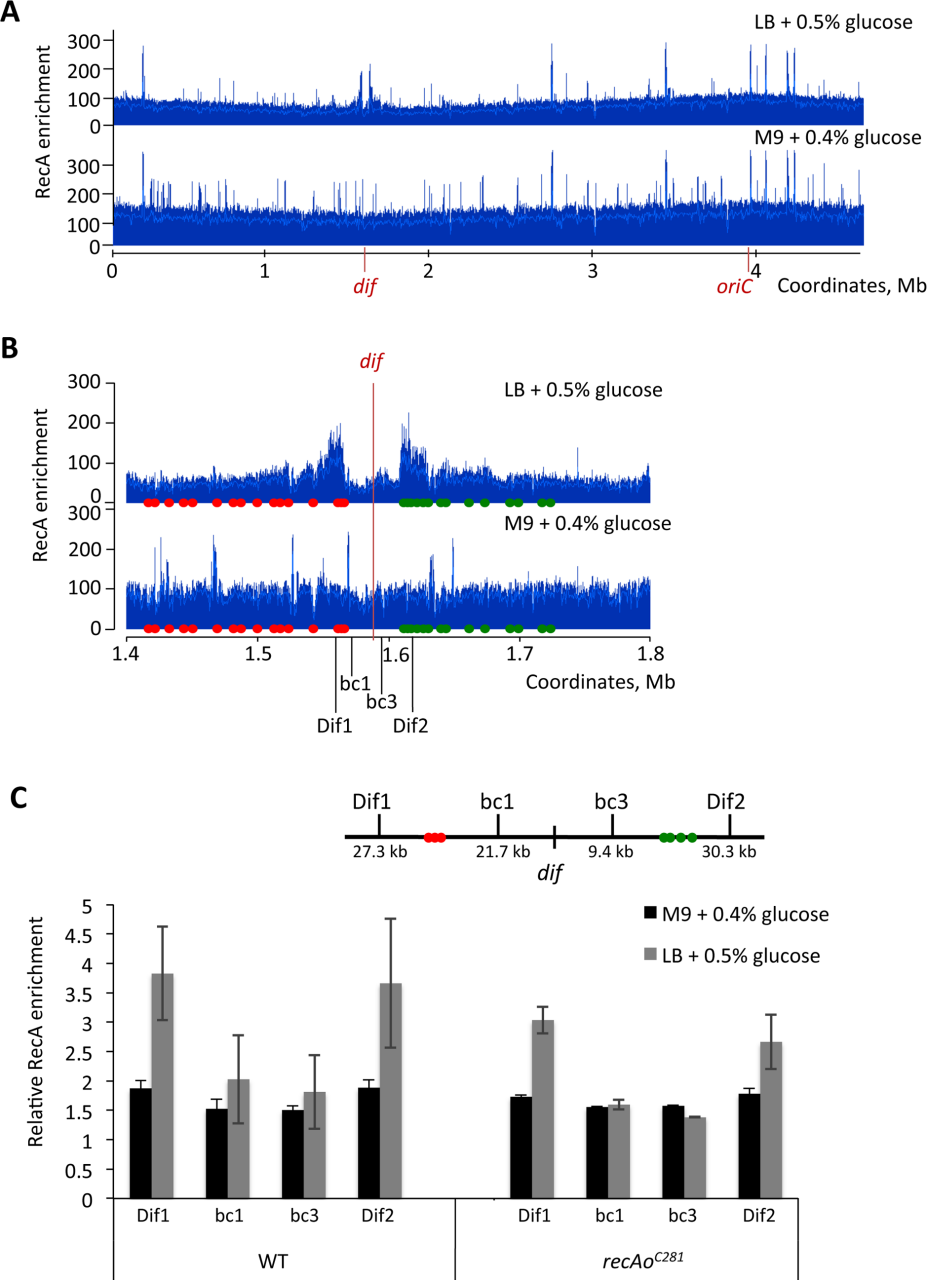
DNA double-strand breaks (DSBs) in the *dif/TerC* region do not occur in RecB^+^ cells in minimal medium (M9). A: Genome-wide RecA binding. RecA-ChIP-seq revealed that the levels of RecA binding at *dif/TerC* region were not increased in a RecBCD^+^ strain grown in M9 minimal medium supplemented with 0.4% glucose. The *dif* site and origin of replication, *oriC*, are indicated. B: Zoom-in to the terminus region of the chromosome. RecA binding corresponds to the positions of correctly oriented Chi sites. The positions of the correctly oriented Chi sites on each side of the *dif* site are shown using red (5’-gctggtgg-3’) and green (3’-ccaccagc-5’) circles. Green Chi sites are oriented in such a way that RecBCD recognises them if it moves left to right on the chromosome from a location close to *dif*. Red Chi sites are recognised by RecBCD that moves in the opposite direction–right to left from a location close to *dif*. The *dif* site is located at 1,588 kb. Positions of qPCR primers (Dif1, bc1, bc3 and Dif2) are indicated. C: ChIP-qPCR of wild-type and *recAo*^*C281*^ strains did not show any increase of relative RecA enrichment in the terminus. RecA enrichment in the terminus is normalised to the background level of RecA enrichment at the *hycG* site. Error bars represent standard error of 3 means. Strains used were wild-type (MG1655) grown in LB medium supplemented with 0.5% glucose and M9 minimal medium supplemented with 0.4% glucose (A, B, C) and *recAo*^*C281*^ grown in LB medium supplemented with 0.5% glucose and M9 minimal medium supplemented with 0.4% glucose (C).

To know whether in wild-type cells RecBCD acts in the *dif/TerC* region, we investigated RecA binding by ChIP followed by qPCR of sequences upstream and downstream of the first Chi site on each side of the *dif/TerC* region (Fig 4, RecBCD loaded at a DSB in the *dif/TerC* region unwinds DNA toward the origin, until it encounters properly oriented Chi sites at which it loads RecA). As previously reported [51] we detected a weak increase of RecA ChIP downstream of Chi when cells were grown in LB, but we did not detect any increase in cells grown in M9 glucose (Fig 4). Similar results were obtained in cells that over-express RecA owing to a mutation in the *recA* gene SOS operator (Fig 4). We conclude that in minimal medium, DNA breakage in the *dif/TerC* region does not occur in RecB^+^ cells, and thus only occurs in *recB* mutants.

### DNA loss in the *dif* region is independent of Topo IV action next to *dif* and of the endonuclease Endo 1

To address the question of the origin of chromosome breaks in the terminus region of a *recB* mutant two enzymes that cleave DNA were tested, Topoisomerase IV (Topo IV) and endonuclease I (Endo I). Topo IV, encoded by the *parC* and *parE* genes, catalyzes the decatenation of daughter chromosomes after replication. Topo IV interacts with both XerC and FtsK, its decatenation activity is stimulated by its interaction with FtsK *in vitro* and a hotspot of activity was detected *in vivo* close to *dif*, which is dependent on its interaction with XerCD [52–55]. We hypothesized that if catenated chromosomes persist in the path of septum closure, an erroneous action of Topo IV during decatenation could be responsible for chromosome breakage. Because the inactivation of Topo IV by a *ts* mutation prevents cell division, and cell division is required for the DSBs studied here (see below), the effects of a *parE10ts* mutation were tested by MFA at 37°C, where Topo IV is impaired but cell division is not affected enough to prevent cell growth [56]. As shown in S4 Fig, the *parE10ts* mutation did not affect DNA loss in the *dif* region at this semi-permissive temperature. Furthermore, interaction of Topo IV with XerCD is required to target its action close to *dif* [55], therefore the lack of effect of *xerC* or *dif* inactivation described above argues against a direct action of Topo IV to break DNA next to *dif*. This was confirmed by using the observation that the Topo IV hotspot next to *dif* is abolished by over-production of the C-terminal region of ParC from a plasmid, (parC-CTD plasmid, [55]): the presence of this plasmid did not affect focus loss (Table 1, S4 Fig). Therefore, the division-dependent DSBs studied here do not result from an erroneous action of Topo IV at *dif*.

The most abundant endonuclease in *E*. *coli* is the periplasmic enzyme Endo 1. We hypothesized that a leak of Endo 1 from the periplasm to the cytoplasm during division might cause cleavage of one chromosome in the terminus region. However, the inactivation of the *endA* gene encoding Endo 1 did not affect the proportion of focus-less cells in a *recB* context (Table 1, S2 Table). The enzyme(s) that introduces the DSBs responsible for DNA loss remain unidentified.

### DNA loss in the *dif* region is dependent on cell division

**Fig 5:**
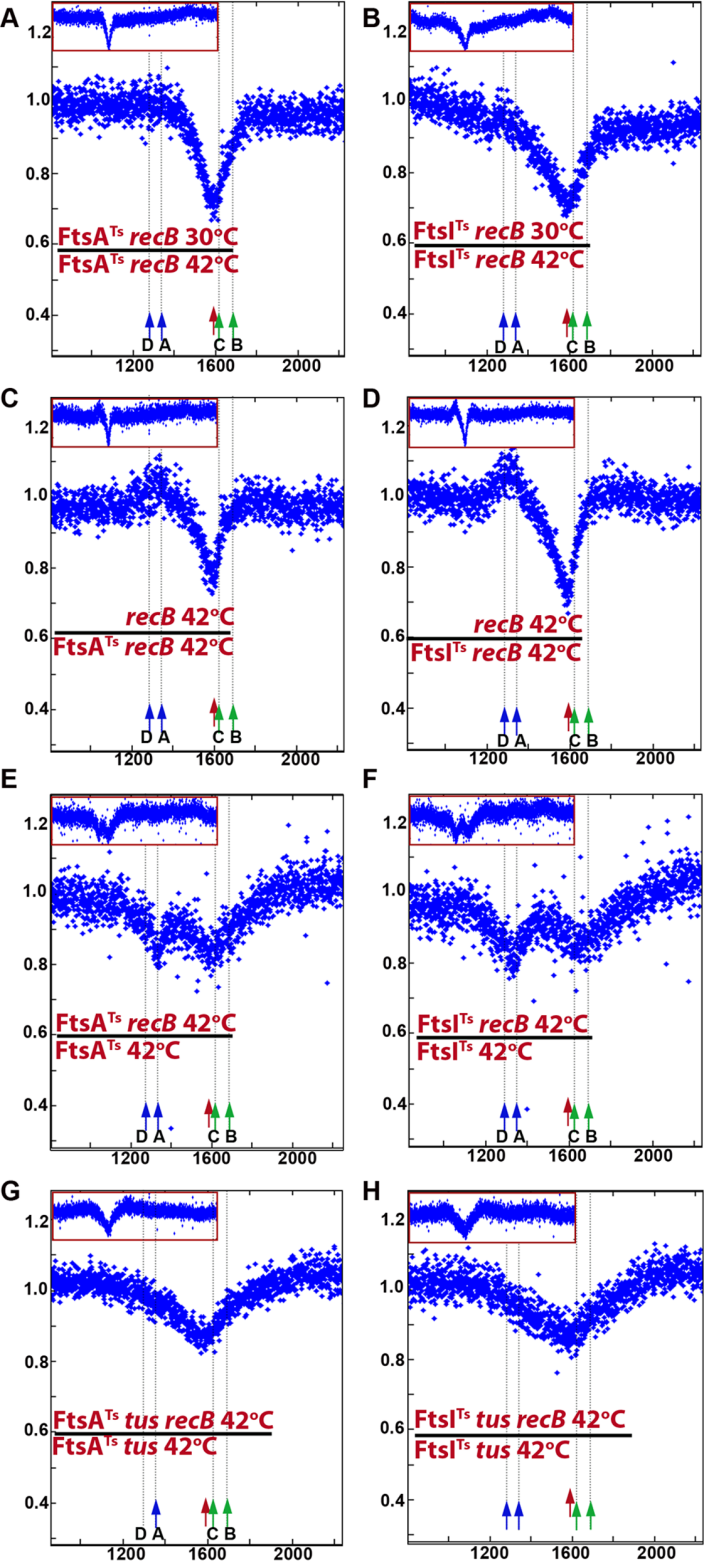
Loss of DNA in the terminus region of *recB* mutant requires cell division. Sequence read frequencies of exponential phase cells normalized to the total amount of reads were calculated for each strain. A and B: Ratios of normalized reads in *fts recB* mutants at 30°C over 42°C are plotted against chromosomal coordinates. The drop of reads ratio in the *dif/TerC* area reflects the reads deficit at 30°C (mainly active cell division) versus 42°C (mainly inactive cell division). C and D: Ratios of normalized reads in *recB* over *fts recB* mutants at 42°C are plotted against chromosomal coordinates. The drop of reads ratio in the *dif/TerC* area reflects the reads deficit in the *recB* mutant at 42°C (active cell division) *versus* the *fts recB* mutants at 42°C (mainly inactive cell division). Note an increased ratio of reads around *TerA/D*, which corresponds to a deficit of *Ter* sequences in *fts recB* mutants (shown in panels E to H). Presumably owing to a partial cell division impediment in the *fts recB* mutants at 30°C, the expected increased ratio is only slightly visible in panel B and not in panel A. E to H: Ratios of normalized reads in *recB* mutants over isogenic RecB^+^ are plotted against chromosomal coordinates. The weak remaining reads deficit in the *dif/TerC* region is *tus* independent and could result from incomplete division blockage by *ftsAts* and *ftsIts* mutations. The reads deficit around *TerA/D* in both *fts* contexts and the reads deficit around *TerB/C* in the *ftsIts* context disappear in the *tus* mutant. Profile ratios of the terminus region are enlarged and profiles of the corresponding entire chromosomes are shown in insets. Original normalized profiles used to calculate ratios are shown in S5 and S6 Figs. The positions of *dif* (red arrow), and of *Ter* sites that arrest clockwise forks (*TerC*, *TerB*, green arrow) and counter-clockwise forks (*TerA*, *TerD*, blue arrow) are shown.

Because time lapse experiments showed that the *ydeV*::*parS*_pMT1_ focus was often lost at the septum and always concomitantly with cell division, we tested whether septum formation plays a role in the loss of the *dif* region. We used three different conditions that affect cell division: *ftsAts* and *ftsIts* thermosensitive mutants, which block an early and a late step of divisome assembly at 42°C, respectively, and cephalexin, a drug that prevents the action of FtsI (reviewed in [24, 25]). Because blocking division produced cells that were highly elongated and difficult to analyse by microscopy, the loss of terminus DNA was examined by MFA. The *ftsAts recB* mutant was compared at 30°C and after 2 hours of incubation at 42°C, to block division (Fig 5A, S5 Fig). Loss of DNA centred on *dif* was observed at 30°C where FtsA is active, but was much weaker at 42°C. A similar result was obtained with the *ftsIts recB* mutant (Fig 5B, S5 Fig). Comparison of a cephalexin-treated with an untreated *recB* mutant showed that cephalexin also prevented DNA loss at *dif* (compare S5 with S1 Fig). Therefore, results in all three cases indicated that loss of DNA in the *dif* region of a *recB* mutant is decreased when septum assembly is prevented. Control experiments showed that DNA loss was similar in a *recB* single mutant at 37°C and at 42°C (S5 Fig). Ratios of *recB* over *fts recB* mutants grown at 42°C revealed two phenomena (Fig 5C and 5D): (i) they confirmed that the loss of reads centred on *dif/TerC* is specific to dividing cells, (ii) they revealed 5–10% more reads in the *TerA/TerD* region in dividing *versus* non-dividing cells. The latter observation suggests that in a *recB* context blocking cell division causes a slight loss of *TerA/TerD* sequences. When results for the *fts recB* cells grown at 30°C and 42°C were compared this increase was not observed (*ftsAts*) or weak at *TerD* (*ftsIts*), presumably because cell division is partially affected in these mutants at the permissive temperature (compare Fig 5A and 5B with 5C and 5D).

Comparisons of RecB^+^ and *recB* mutants in *ftsIts* (or *ftsAts*) contexts at 42°C, revealed two regions of DNA loss caused by *recB* inactivation, weaker than in dividing cells and centred on *TerA* and on the *dif/TerC* region (Fig 5E and 5F). The effects of *recB* inactivation in *ftsIts tus* and *ftsAts tus* mutants were analyzed (Fig 5G and 5H). The small peak in the *TerA* region disappeared in the *tus* context, showing that it results from replication arrest at *TerA* (compare Fig 5G and 5H with Fig 5E and 5F, S6 Fig). Accordingly, in the *ftsIts* context the weak *recB*-dependent DNA loss in the *TerB*/*TerC* region was displaced to the *dif* region when *tus* was inactivated. The peak of DNA loss at *dif* in *fts tus recB* mutants is weaker than in dividing cells (compare Fig 5G and 5H with Fig 2B and 2D), accounting for the difference between dividing and non-dividing cells shown above (Fig 5A, 5B, 5C and 5D). We conclude from these experiments that (i) DNA loss around *dif* in the *recB* mutant is decreased by inactivating cell division (Fig 5A–5D), and (ii) weaker peaks of DNA loss that require the Tus protein can be observed at *TerA* in non-dividing *recB* mutants (Fig 5E–5H).

### DNA loss occurs at a new GC skew convergence zone created by a terminus deletion

**Fig 6:**
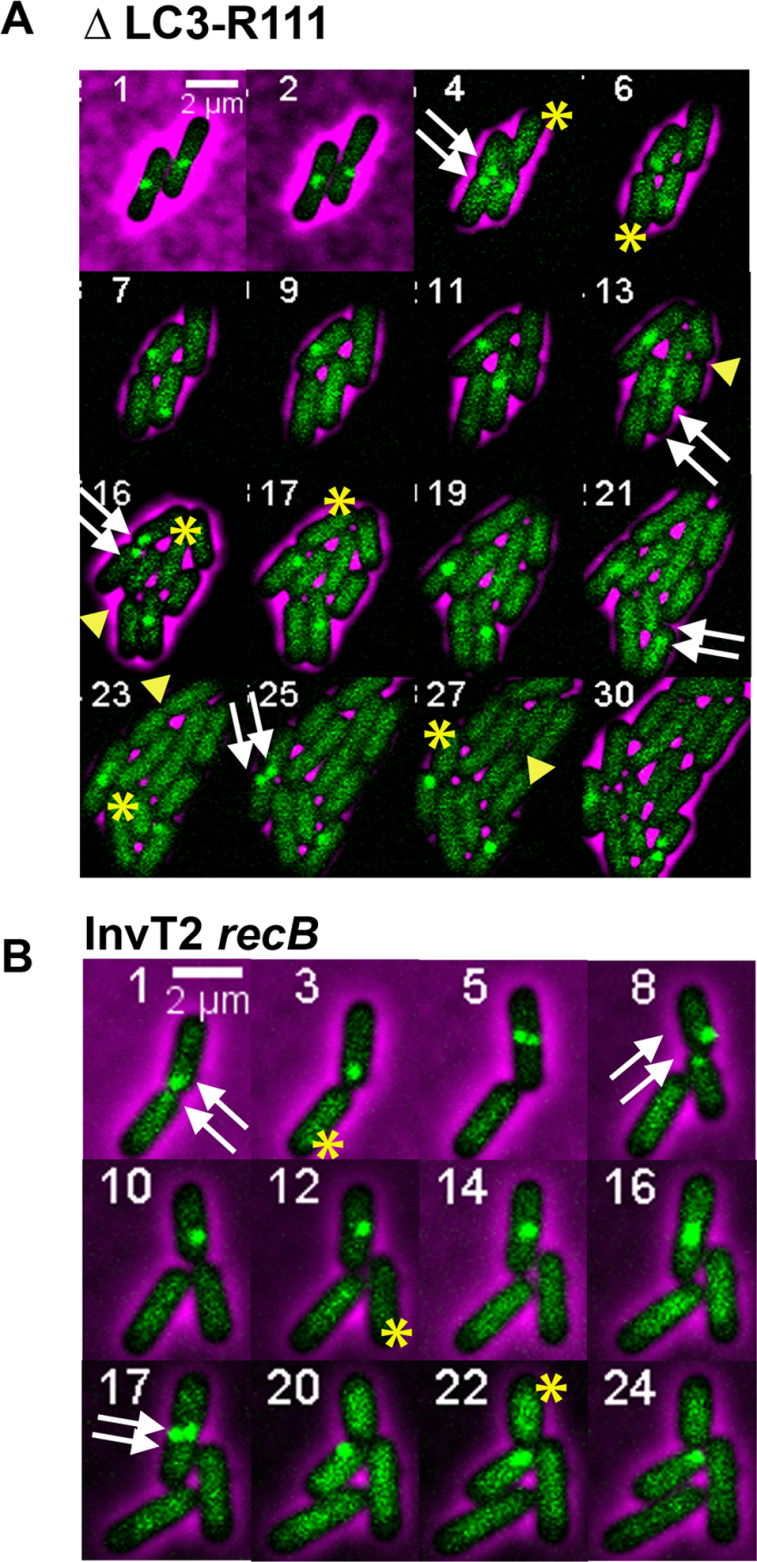
Micrographs showing examples of focus loss during growth of ΔLC3-R111 *recB* and InvT2 *recB* mutants. Time lapse experiments were carried out as in Fig 2. The double white arrows indicate the presence of two foci before division, the yellow stars show cells that have no focus following division. A. In the ΔLC3-R111 *recB* mutant, two examples of hereditary focus loss in one daughter cell are shown (single yellow star). In addition, focus-less cells divide owing to the deletion of the *hipAB* genes (yellow arrowhead), but for fewer generations than in the *dif hipA* mutant because the large deletion brings the first essential genes closer to the site of breakage. B. In the InvT2 *recB* mutant an example of hereditary focus loss in one daughter cell is shown (single yellow star).

To further test whether the site of convergence of GC skew determines the localisation of DNA loss, we constructed mutants in which a new GC skew convergence zone was created in the terminus region. First, we used a strain where the entire terminus region from 1 379 810 to 1 617 226 was deleted (ΔLC3-R111 strain, Fig 1C). This 237 kb deletion removes half of the DNA region degraded in the *recB* single mutant including *dif*, *hipA* and *TerC*. It defines a new 102 kb replication fork trap between *TerA* and *TerB* and creates a new GC skew converging zone at the junction, next to which we inserted a *parS* site (*pspE*::*parS*_pMT1_). Because the ΔLC3-R111 mutant lacks the *dif* site, it showed 18% of focus-less cells. In the ΔLC3-R111 *recB* mutants 49% of cells were devoid of a *pspE*::*parS*_pMT1_ focus (Table 2). Time lapse analyses showed that the loss of a *pspE*::*parS*_pMT1_ focus in the ΔLC3-R111 *recB* resulted mostly from loss of one focus in one daughter cell at the time of division as in the original *recB* mutant (Fig 6A); we counted 19% initial events and 77% of them were transmitted to progeny (Table 2). Focus loss also occurred at a lower frequency in both daughter cells at the time of division, presumably resulting from dimer breakage (Fig 6A). The ΔLC3-R111 deletion removes *dif* and *hipA*, but shortens the region devoid of essential genes that can be degraded without preventing cell propagation. Accordingly, we observed one or two divisions of focus-less cells owing to the absence of *hipAB*.

**Table 2:** The sequence close to the new chromosome arms junction is lost in the ΔLC3-R111 mutant.

*pspE*:: *parS*_pMT1_	% cells with 0 focus	initial events	transmitted
*recB*	15.6 ± 2.7		
ΔLC3-R111	16.6 ± 1.9		
ΔLC3-R111 *recB*	49.2 ± 2.5	19% (295)	77.1%
ΔLC3-R111 *tus*	18.4 ± 1.4		
ΔLC3-R111 *recB tus*	60.5 ± 3.5		

**Fig 7:**
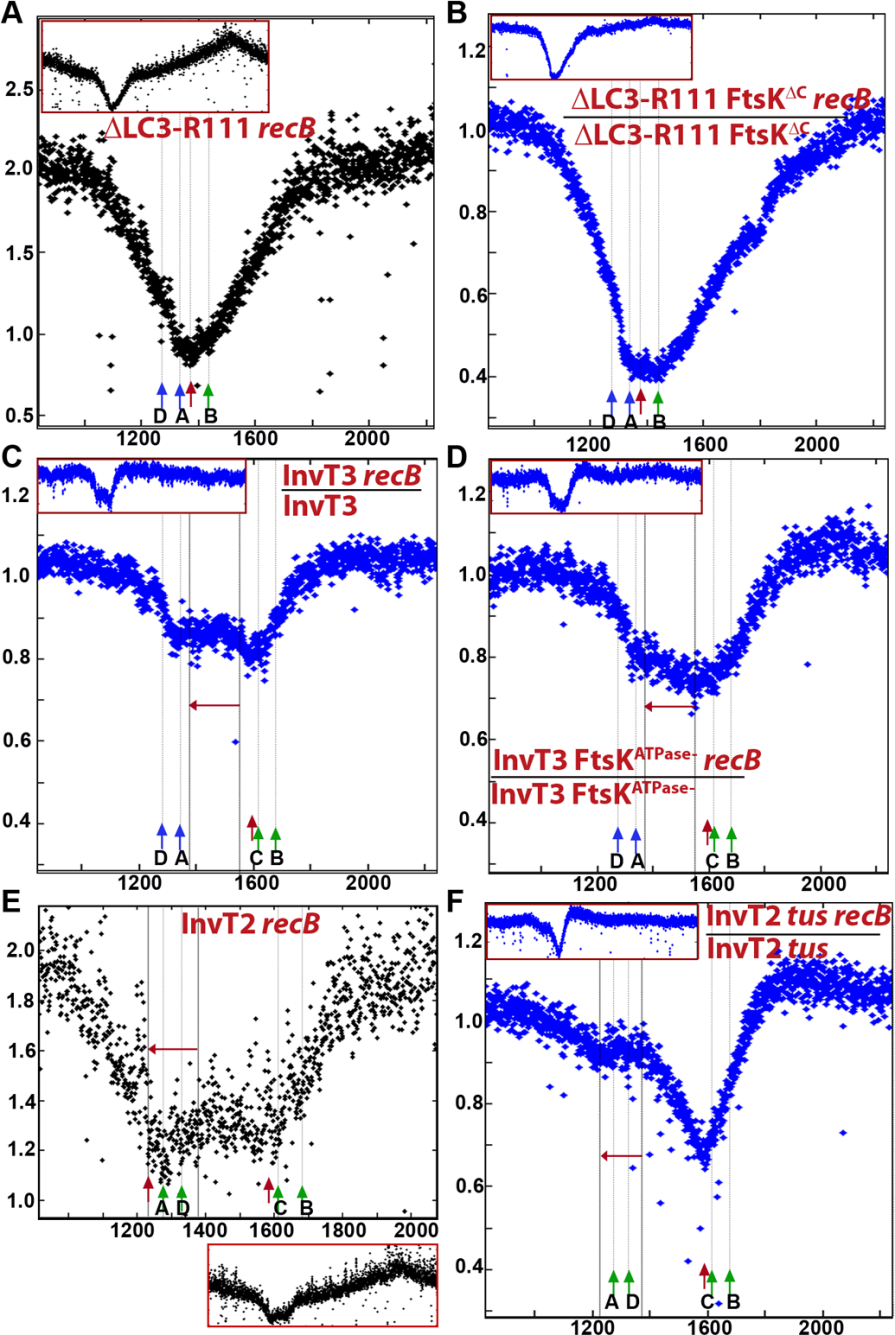
Loss of DNA in the terminus region of *recB* mutant is independent of DNA sequence and is unaffected by inversion of an adjacent sequence. Sequence read frequencies of exponential phase cells normalized to the total amount of reads were calculated for each strain. For the ΔLC3-R111 *recB* (A) and InvT2 *recB* (E) mutants, normalized numbers of reads are directly plotted against chromosomal coordinates. For the other mutant strains, ratios of normalized reads in isogenic RecB^+^ and *recB* mutants are plotted against chromosomal coordinates. ((B) ΔLC3-R111 *ftsK*^Δ*CTer*^, (C) InvT3, (D) InvT3 *ftsK*^ATPase^, (F) InvT2 *tus*). Profile ratios of the terminus region are enlarged and profiles of the corresponding entire chromosomes are shown in insets. Original normalized profiles used to calculate ratios are shown in S7, S8 and S9 Figs. The position of *dif* in InvT2 and InvT3, or of convergence of GC skew in ΔLC3-R111, is indicated by a red arrow. *Ter* sites that arrest clockwise forks (*TerC/TerB* in all strains, *TerA/TerD* in InvT2, green arrow) and counter-clockwise forks (*TerA*, *TerD*, except in InvT2, blue arrows) are shown.

For unknown reasons the MFA analysis of the ΔLC3-R111 chromosome (S7 Fig), showed a breakpoint in the read copy number around *TerA*, which was not detected in the *recB* mutant (Fig 7A). The ratio of reads in *recB* mutant over RecB^+^ cells is affected by this breakpoint, and in Fig 7A we present directly the MFA result of the ΔLC3-R111 *recB* mutant. The peak of DNA loss measured by MFA was located at the new junction of the chromosome arms, about 65 kb from *TerB* (Fig 7A). Similarly to the lack of effect of *tus* inactivation on DNA loss in the *recB* mutant (Table 1), inactivating *tus* in the ΔLC3-R111 *recB* mutant did not prevent DNA loss at the GC skew convergence point (Table 2). For unknown reasons, inactivation of the DNA translocation activity of FtsK in the ΔLC3-R111 mutant led to an amplification centred on the midpoint between *TerA/D* and *TerF* and the breakpoint between *TerA* and *TerB* was not detectable (ΔLC3-R111 *ftsK*^Δ*CTer*^ S7 Fig). However, DNA loss occurred in ΔLC3-R111 *ftsK*^Δ*CTer*^ *recB* as in ΔLC3-R111 *recB*, and the inactivation of FtsK translocase slightly widened the maximum point of DNA loss, to the entire 105 kb region between *TerB* and *TerA* (Fig 7B), These results show that the position of division-induced DSBs is determined by the point of GC skew convergence, in a way that is independent of the sequence of this junction, and is more precisely targeted to the KOPS convergence point in the presence of the FtsK translocation activity.

### DNA loss remains at *dif* when an adjacent region is inverted

We then created a new GC skew convergence zone by inverting a region of the terminus. In the InvT3 mutant, a ~175 kb sequence is inverted on the right chromosome arm, which does not contain any *Ter* site and starts about 34 kb from *dif* (Fig 1B). In this strain the main GC skew convergence zone is moved 209 kb to the left of *dif*, and the *dif* position becomes a minor convergence zone with on its left only 34 kb of DNA in the original orientation. InvT3 and InvT3 *recB* strains were compared by MFA (Fig 7C, S8 Fig). Inactivation of *recB* in InvT3 created a new degraded region corresponding to the entire inverted sequence, but no peak of DNA loss at the new convergence zone. Importantly, the main DNA degradation peak in the *dif* region was still present (Fig 7C, S8 Fig). Microscopy analysis confirmed a specific loss of the *dif* region by showing that the proportion of *ydeV*::*parS*_pMT1_ focus-less cells was similar in InvT3 *recB* and *recB* mutants (38% and 32% respectively, Table 1, S2 Table; the additional focus-less cells observed in InvT3 RecB^+^ could result from a perturbation of segregation because of KOPS inversion, causing irreparable damage). The inactivation of the ATPase function of *ftsK* enlarged the degraded region but the maximum of DNA loss was still in the *dif* region (Fig 7D). Comparison of FtsK^+^ and *ftsK* mutant MFA in the *recB* context suggests that FtsK-mediated translocation slightly protects the inverted region from degradation. It is also interesting to note that in the absence of the FtsK translocation activity, loss of the reads in the 1380–1554 kb region in the *recB* mutant was higher when this sequence was inverted than when it is in the original orientation (compare Fig 7D with Fig 3C). This observation suggests the existence of a system other than FtsK able to detect the sequence orientation. Nevertheless, these results also indicate that a 175 kb GC skew convergence zone is not sufficient to create a division-induced DSB.

In the InvT2 mutant a 150 kb region encompassing the *TerA* and *TerD* sites and located 209 kb from *dif* is inverted (Fig 1B). Clockwise replication forks are expected to be arrested in the *TerA-TerD* region in this mutant, and the replication fork trap is moved between the inverted *TerA* and *TerE*. Accordingly, the MFA profile of the InvT2 mutant shows that replication forks meet in the *TerA-TerD* region, ~250–300 kb away from *dif* (S9 Fig). As for the LC3-R111 mutant, direct results of the InvT2 MFA are shown in Fig 7E because the breakpoint of read copy number around TerA affects the ratio of RecB^+^ *versus recB* mutant reads (Fig 7, S9 Fig). Two peaks of DNA degradation were clearly detected by MFA in the InvT2 *recB* mutant: the one centred at *dif* observed in all *recB* mutants and a new one coincident with the inverted *TerA* site (Fig 7E, S9 Fig). The inactivation of *tus* suppressed the *TerA*-associated degradation but not DNA loss at *dif*, and allowed the detection of some DNA degradation associated with the inversion region, as in InvT3 (Fig 7F, S9 Fig). The observation that the InvT2 inversion did not affect inheritable division-dependent focus loss was confirmed by microscopy, as the proportion of *ydeV*::*parS*_pMT1_ focus-less cells increased in InvT2 from 8% to 42% upon *recB* inactivation (Table 1, Fig 6B). The observation of Tus-dependent DNA loss at TerA confirms that DNA breakage occurs at an artificially introduced *Ter* site that creates a new replication fork trap, as observed with TerB* (Fig 2C and 2D). In addition, these results confirm that division-induced DSBs in the *dif* region are not affected by the creation of a new GC skew convergence zone, as observed with InvT3. Furthermore, in InvT2 as in InvT3 the new DNA convergence zone does not show a peak of DNA loss, in contrast with ΔLC3-R111, but the number of reads in the whole inverted region is lower than when this sequence is not inverted (compare Fig 7C with Fig 2A and Fig 7F with Fig 2B).

## Discussion

**Fig 8:**
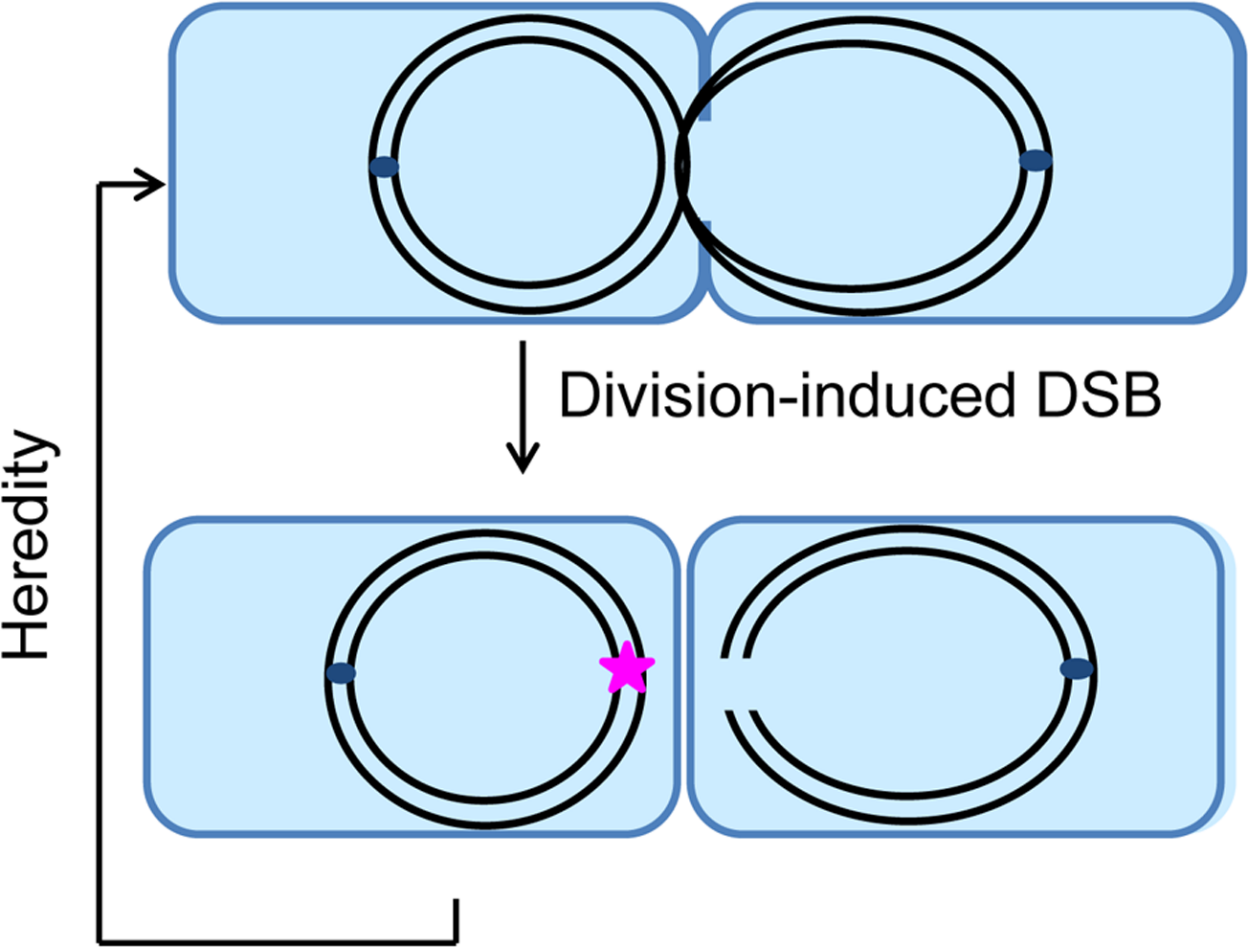
Schematic representation of terminus DNA loss. Terminus DNA loss occurs after the two chromosomes are fully replicated. The *dif/TerC* region of one of the two chromosomes remains in the path of the septum and is broken during cell division (right cell). The other chromosome carries a scar that leads to the transmission of the reaction to the progeny (left cell). This representation of our results lacks details because we do not know at present the molecular mechanism that triggers these events in the *recB* mutant. Blue rectangle: *E*. *coli* cells, black lines: chromosome DNA strands, dark blue little circles: replication origins, pink star: scar carried by the unbroken chromosome.

In the present study, we reveal that the DNA region of the *E*. *coli* chromosome terminus, previously shown to be under-represented in the *recB* mutant [34, 35], is lost following division-dependent chromosome breakage (Fig 5). We have demonstrated that DNA loss in the *dif* region of *recB* mutants occurs with the following characteristics: (i) after duplication of the region, (ii) at the time and often at the site of cell division, (iii) in one of the two daughter chromosomes, and (iv) is transmitted to progeny (Figs 2, 3 and 6 and S1–S6 Videos). This DNA loss is independent of the position of replication termination, as we observed that DNA loss at *dif/TerC* is unaffected in a *tus* mutant, or when replication forks are prevented from reaching this region by *pspE*::TerB* or by the inversion of *TerA*-*TerD* in the InvT2 mutant (Figs 2, 6 and 7, Table 1). It occurs in the absence of chromosome dimer resolution (not affected in *dif* and *xerC* mutants, Fig 3, Table 1). In the absence of FtsK-driven DNA translocation, terminus DNA loss is less precisely targeted to the KOPS convergence sequence (Fig 3 and Fig 7), but follows the same pattern as in FtsK^+^ cells (Table 1, S5 Video). DNA loss can occur at least at two different GC skew convergence zones regardless of their sequence (*recB* and ΔLC3-R111 *recB*, Figs 6 and 7, Tables 1 and 2), but DNA loss at the natural GC skew convergence point is not affected by a nearby 150–175 kb inversion (InvT2 and InvT3 inversions, Figs 6 and 7, Table 1). The only mutations that strongly decrease terminus DNA loss in a *recB* context are those that block cell division (*ftsAts* and *ftsIts* mutants at 42°C, cephalexin treatment at 37°C; Fig 5). A schematic representation of terminus DNA loss according to our results is shown in Fig 8.

### Chromosome breakage occurs at the time of cell division and requires division

Septum-induced breakage was previously reported in *xer* and *dif* mutants, in which chromosome dimers are not resolved to monomers and remain in the path of the closing septum [46]; as expected we observed dimer breakage in our experiments, which occurs specifically in mutants affected for dimer resolution (*xer*, *dif*, *ftsK*) and is characterized by a loss of *ydeV-parS*_pMT1_ foci in both daughter cells along with a significant delay in cell division. Dimer breakage during septum formation was called guillotining, a term that does not describe precisely the molecular events leading to DNA DSBs. If we assume that chromosome dimer breakage results from physical tension associated with the pulling of two linked chromosomes during segregation, then the breakage of one chromosome observed in *recB* cells implies that this chromosome is broken as a consequence of being attached in the terminus region while the origin is gradually pulled towards the daughter cell. This attachment could be a covalent link with the other daughter chromosome after replication completion, or a strong binding to a septum protein. It is unlikely that this link is topological, since DNA loss is unaffected in conditions that perturb Topo IV action. It is noticeable that breakage occurs without any delay in cell division, in contrast with dimer breakage. Alternatively, breakage could be enzymatic, but the nature of the nuclease remains mysterious.

Importantly, we did not detect any focus loss in the *recB* mutant at any other time point in the cell cycle than cell division, and our measures of replication speed based on the MFA results show that the *recB* mutation does not affect replication progression (the ratio of *recB* versus wild-type reads is constant all along the chromosome except at the terminus and equal to 1 in Fig 2A). This is in agreement with the recently published results, where authors using flow-cytometry analysis concluded that absence of RecB does not affect chromosome replication speed [57]. Hence, we propose that the main source of chromosome breakage in the *recB* mutant grown in M9 is not replication fork impediments but rather division-induced breaks in the terminus region of the chromosome.

### Broken chromosomes are degraded

Following chromosome breakage, degradation of the DNA double-stranded ends by exonucleases is responsible for DNA loss. This step was postulated but never demonstrated [34, 35], and so far the formal possibility of under-replication of the *dif* region being responsible for the low number of reads observed in MFA experiments could not be excluded. In time lapse experiments, we most often see two *ydeV-parS*_pMT1_ foci before one of them disappears, which ascertains for the first time the presumed assumption that DNA loss does not result from a lack of replication but from DNA degradation of a replicated chromosome. Furthermore, the *dif hipA* and the ΔLC3-R111 mutants behaved as expected *i*.*e*., in the absence of HipA-induced cell death, the broken chromosomes are slowly degraded and cells with a broken chromosome propagate until degradation by exonucleases reaches essential genes.

### DNA loss occurs specifically in the GC skew convergence region

DNA loss occurs at the site of GC skew convergence, and is observed at the new GC skew convergence zone in the strain that carries a large terminus deletion, confirming that the phenomenon is not DNA sequence specific. In addition, DNA loss is not affected by replication orientation, which progresses across *dif* in the clockwise direction in the majority of wild-type cells [7], but not when replication is arrested prior to *dif* by an ectopic TerB* site (*pspE*::*TerB*) or by inverting *TerA* and *TerD* (InvT2). We have shown that division-induced chromosome breakage is independent of any specific DNA sequence. These observations support a model in which the chromosome terminus region is somehow specifically and precisely positioned in the path of the division machinery. This positioning is more centred on the KOPS convergence zone when FtsK translocation is active, but remains centred on *dif* in FtsK mutants, and therefore relies on a so far unknown process. Division-induced chromosome breakage occurs in a sub-population of dividing *recB* cells when the positioning is inappropriately controlled. In addition to causing breakage of one chromosome, the improper processing of the terminus leaves a mark on the intact chromosome, which is responsible for the transmission of the defect to the next generation.

### *Ter*-induced DNA DSBs

In addition to the septum-induced DNA DSBs described above, we observed a Tus-dependent loss of reads, suggesting DNA breakage in specific *recB* mutant conditions: (i) at ectopic or inverted Ter sites (*pspE*::*TerB* Fig 2C and 2D, *TerA* in InvT2 Fig 7E and 7F) and (ii) in the *TerA-TerD* region of cell division mutants (*ftsAts* and *ftsIts* at 42°C, cephalexin treated cells, Fig 5). The loss of reads around *Ter* sites is symmetrical, indicating that DNA breakage does not occur after blockage of the first fork that reaches *Ter* (only the replicated side of *Ter* would then be degraded). Previous studies showed that forks blocked at ectopic *Ter* sites are stable, and that DNA double-strand ends are formed at such *Ter* sites upon arrival of a second round of replication behind the first blocked one, by rear-ending, but in these previous mutant strains fork-merging was prevented [58, 59]. The Tus-*Ter* specific DNA breaks observed in the present work could therefore result from abnormal replication forks merging at *Ter* sites. However, although our MFA experiments are only semi-quantitative, Tus-dependent DNA loss at *Ter* sites seems to occur in a lower proportion of cells than division-induced breaks, with a weaker peak of DNA loss than the peak of division-induced DNA loss. Furthermore, the absence of DNA loss at *TerA* in wild-type cells shows that Tus-*Ter* induced breaks do not occur at a natural *Ter* site in cells that divide normally. The observation that blocking cell division triggers Tus-dependent DNA loss at *TerA* suggests an unknown link between replication fork merging and cell division, a link which would also be perturbed in dividing cells by arresting forks at an ectopic *Ter* site. Our observation suggests that replication termination at *TerC*, or forks merging at other sequences than *Ter* sites, is the most favourable condition during normal cell division and any change in this arrangement leads to loss of DNA at the new active *Ter* site. Further work will be needed to understand how *Ter*/Tus dependent DNA DSBs are made, and whether a common mechanism is involved upon division blockage at the natural *TerA* site and in dividing cells at ectopic *Ter* sites.

In conclusion, we have shown that DNA degradation in the GC skew convergence region occurs in a subpopulation of *recB* growing cells. The reaction is transmitted to progeny and is strongly decreased when cell division is prevented. It is targeted to the KOPS merging zone by the translocase activity of FtsK, and occurs in a broader chromosome terminus region in mutants that lack this activity. Since our time-lapse experiments did not show any growth defect or loss of focus in the *recB* mutant at any other time than cell division, we propose that division-induced DNA breakage could be responsible for the decreased viability of *recB* cells under normal laboratory growth conditions. These findings open new fields of investigation in search for the molecular mechanism responsible for this reaction.

## Materials and methods

### Strains and plasmids

All *E*. *coli* strains are derivatives of MG1655. Strains and plasmids are described in S1 Table. MM is M9 [60] supplemented with 0.4% glucose. Standard transformation and P1 transduction procedures were as described [60]. *pspE*::*TerB*-Cm^R^, *endA*::Kan^R^, *araC*::*parB*_pMT1_-Cm^R^, *araC*::*parB*_pMT1_-Apra^R^, *ydeV*::*parS*_pMT1_-Apra^R^ mutations were constructed by gene replacement (recombineering) as described in [61], using DY330 [62]. All other strains were constructed by P1 transduction. All mutations introduced by P1 transduction were checked by PCR and all new mutations constructed by recombineering were checked by PCR and sequencing. *recB* mutations were tested by measuring UV sensitivity. Deletion ΔR111-LC3 and inversions InvT2 and InvT3 were made as described [63].

The *araC* open reading frame was replaced by the *yGFP*-*parB*_pMT1_-Cm^R^ sequence, to express yGFP-ParB protein under the control of the constitutively expressed *araC* promoter. For this construction, the Cm gene was amplified from pKD3 plasmid using primers harboring an *Hind*III site (S3 Table). Amplified fragments were digested with *Hind*III and cloned into the *Hind*III site of pFHC2973 to make pFHC2973- *yGFP*-*parB*_pMT1_-Cm^R^ plasmid (Nielsen et al., 2006). Clones were confirmed by PCR and sequencing using flanking primers. The *yGFP*-*parB*_pMT1_-Cm fragment was then amplified from pFHC2973- *yGFP*-*parB*_pMT1_-Cm^R^ plasmid using primers 582 and 583 (S3 Table) and inserted downstream of the *araC* promoter by the gene replacement method (recombineering) as described in [61], using DY330 [62].

### Marker frequency analysis

Cells were grown in M9 minimal medium supplemented with 0.4% glucose to exponential phase (0.2 OD 650 nm). Chromosomal DNA was extracted using the Sigma GenElute bacterial genomic DNA kit. 5 μg of DNA were used to generate a genomic library according to Illumina's protocol. The libraries and the sequencing were performed by the High-throughput Sequencing facility of the I2BC (http://www.i2bc.paris-saclay.fr/spip.php?article399&lang=en, CNRS, Gif-sur-Yvette, France). Genomic DNA libraries were made with the ‘Nextera DNA library preparation kit’ (Illumina) following the manufacturer’s recommendations. Library quality was assessed on an Agilent Bioanalyzer 2100, using an Agilent High Sensitivity DNA Kit (Agilent technologies). Libraries were pooled in equimolar proportions. 75 bp single reads were generated on an Illumina MiSeq instrument, using a MiSeq Reagent kit V2 (500 cycles) (Illumina), with an expected depth of 217X. An in-lab written MATLAB-based script was used to perform marker frequency analysis. Reads were aligned on the *Escherichia coli* K12 MG1655 genome using BWA software. Data were normalized by dividing uniquely mapping sequence reads by the total number of reads. Enrichment of uniquely mapping sequence reads in 1 kb non-overlapping windows were calculated and plotted against the chromosomal coordinates.

### Fluorescence microscopy

Cells were grown in M9 minimal medium supplemented with 0.4% glucose to exponential phase (0.2 OD 650 nm) and spread on a 1% (wt/vol) agarose pad for analysis. For snap-shot analyses, cell images were acquired using a DM6000-B (Leica) microscope with MetaMorph software (Version 7.8.8.0, Molecular Devices) and analyzed using ImageJ. Images were taken from 5–10 different fields in each experiment. Two to three independent experiments were carried out to calculate mean and standard deviation for distributions of foci for each strain. For time-lapse analyses, 0.4% glucose agarose pads were used, the slides were incubated at 30°C and images were acquired every 10 minute by an Evolve 512 electron-multiplying charge-coupled device (EMCCD) camera (Roper Scientific) attached to an Axio Observe spinning disk (Zeiss). Image acquisition was done using MetaMorph software (Version 7.8.11.0, Molecular Devices). At each time point, we took a stack of 32 bright-field images covering positions 1.6 μm below and above the focal plane. Image acquisition was performed on five selected different fields corresponding to different cell populations in each experiment. Final images were reconstructed from image stacks using an in-lab written MATLAB-based script. Image analysis was done manually using ImageJ software. For each mutant strain analyzed, two independent time-lapse experiments were realized, each providing five images with 5–10 bacteria per image at the start. The number of divisions that provided two foci-containing cells and the number of first divisions that provided one focus-containing and one focus-free cells were manually counted. Only cells that started with a normal division were taken into account (few cells produced a focus containing-cell and a focus-less cell from the start and were not counted as initial events, as they did not show any normal division preceding the initial event). The percentage of initial events (between parentheses in Table 1) corresponds to the ratio of cell divisions where a focus is lost in one daughter cell for the first time to the total number of cell divisions. For example, in the scheme shown in Fig 2E, we counted 2 initial events (#2 and 7) out of 9 total cell divisions, and 100% heredity.

### ChIP sample preparation

Cells were grown in either M9 minimal medium supplemented with 0.4% glucose, 5 μM CaCl_2_ and 1 mM MgSO_4_ or LB medium supplemented with 0.5% glucose at 37°C as described in Cockram et al, 2015 [51].

#### Library preparation for high-throughput sequencing and ChIP-seq data analysis

Input and ChIP samples were processed as described in [51]. Adaptor-ligated DNA was size-selected with an average size of ~350 bp Samples that were grown in LB medium were sequenced using 50 bp single-end sequencing on the Illumina HiSeq 2500 platform and samples that were grown in M9 medium were sequenced using 75 bp paired-end sequencing on the Illumina HiSeq 4000 platform. ChIP-seq data analysis was performed as described in Cockram et al, 2015.

#### ChIP-qPCR

All real-time quantitative PCR reactions were performed as described in [51]. All qPCR primers are listed in S3 Table.

## Supporting information

S1 Fig Marker frequency analysis of exponential phase cells. Original normalized profiles are shown except for the top middle panel where ratio of *recC* and wild-type data is shown.
(TIF)

S2 Fig Marker frequency analysis of exponential phase cells. (TIF)

S3 Fig Marker frequency analysis of exponential phase cells. Original normalized profiles are shown except for the bottom panel where ratio of *Fts* ^*ATPase*^ and wild-type data is shown.
(TIF)

S4 Fig Marker frequency analysis of exponential phase cells. Original normalized profiles are shown except for the top left panel which shows ratio of *parEts recB* and wild-type, and top right panel which shows the ratio of *recB* containing the indicated plasmids.
(TIF)

S5 Fig Marker frequency analysis of exponential phase cells. Original normalized profiles are shown except for the lowest right panel where ratio of cephalexin-treated *recB* and wild-type cells is shown.
(TIF)

S6 Fig Marker frequency analysis of exponential phase cells. (TIF)

S7 Fig Marker frequency analysis of exponential phase cells. Original normalized profiles are shown. Lower left panel shows the magnified terminus region of ΔLC3-R111 mutant. In ΔLC3-R111 the two replication forks are expected to merge at equal distance from the origin in both directions, between *TerA* and *TerB*. However, the MFA shows an excess of reads in the region on the left of *TerA* on the figure compared to the region on the right, with a breakpoint around *TerA*, which suggests some DNA amplification left of *TerA* in a RecB^+^ context. There is no evidence for this amplification phenomenon in a *recB* mutant context (Fig 7A) and for this reason we present the results obtained in the ΔLC3-R111 *recB* mutant and not the ratio of ΔLC3-R111 *recB* to ΔLC3-R111 RecB^+^ in Fig 7A. Note that the breakpoint in the number of sequence reads around *TerA* is not detected in the FtsK^ΔCTer^ context, where instead an unexplained amplification is apparent between *TerB* and *TerF*. Further work will be needed to fully understand these phenomenon.
(TIF)

S8 Fig Marker frequency analysis of exponential phase cells. (TIF)

S9 Fig Marker frequency analysis of exponential phase cells. Original normalized profiles are shown. Lower left panel shows the magnified terminus region of InvT2.
(TIF)

S1 Table Strain list. (PDF)

S2 Table Percentage of cells with zero, one or two foci in mutant strains. (PDF)

S3 Table Oligonucleotides used in this study. (PDF)

S1 Video Time lapse microscopy of *recB* cells. Cells were mounted on M9 glucose agarose pad and incubated at 30°C on stage of the microscope. Images were captured every 10 min. *dif/TerC* region of chromosome is visualized as green fluorescence focus by binding of GFP-ParBpMT1 protein to *ydeV*::*parS*pMT1. All frames are labelled. The double white arrows indicate visualization of two foci before division, the yellow stars show cells that have lost focus following division. Note that the focus-less cells do not divide further while the sister cell that has kept the *ydeV*:: *parS*pMT1 locus grows, divides and produces cells without focus for subsequent generations.
(AVI)

S2 Video Time lapse microscopy of *recB* cells. (AVI)

S3 Video Time lapse microscopy of *tus recB* cells. (AVI)

S4 Video Time lapse microscopy of *dif* cells. In contrast to *recB* cells, where only one daughter cell loses focus, in some *dif* cells both daughter cells lose focus due to breakage of unresolved chromosome dimers during cell division (Frame 26). Importantly, there was a considerable delay in cell division observed before the loss of focus in *dif* cells (Frame 17–26).
(AVI)

S5 Video Time lapse microscopy of *ftsK*^ΔC*Ter*^ *recB* cells. In addition to the *recB* phenotype (Frame 16, 26 etc.), where only one daughter cell loses focus, in *ftsK*^ΔC*Ter*^ *recB* cells, occasionally, both daughter cells lose focus due to breakage of DNA in unresolved chromosome dimers during cell division (Frame 31).
(AVI)

S6 Video Time lapse microscopy of *ftsK*^ΔC*Ter*^ *recB* cells. In this example we show that in addition to *recB* phenotype (Frame 5, 15 and 24), where one daughter cell loses focus at the time of cell division, in *ftsK*^ΔC*Ter*^ *recB* cells, foci could also sometimes disappear randomly during the cell cycle (Frame 32). This unusual loss is indicated by a yellow cross.
(AVI)

S7 Video Time lapse microscopy of *ftsK*^ΔC*Ter*^ *recB* cells. In this example we show that some *ftsK*^ΔC*Ter*^ *recB* cells lose focus and die due to other problems, which may arise because of the inhibition of FtsK translocation and need of RecB for repair.
(AVI)
