# Supplementary figures and images for: Division-induced DNA double strand breaks in the chromosome terminus region of Escherichia coli lacking RecBCD DNA repair enzyme
Source: PLoS Genet. 2017 Oct 2;13(10):e1006895. doi: 10.1371/journal.pgen.1006895 (PMC5638614; doi:10.1371/journal.pgen.1006895)

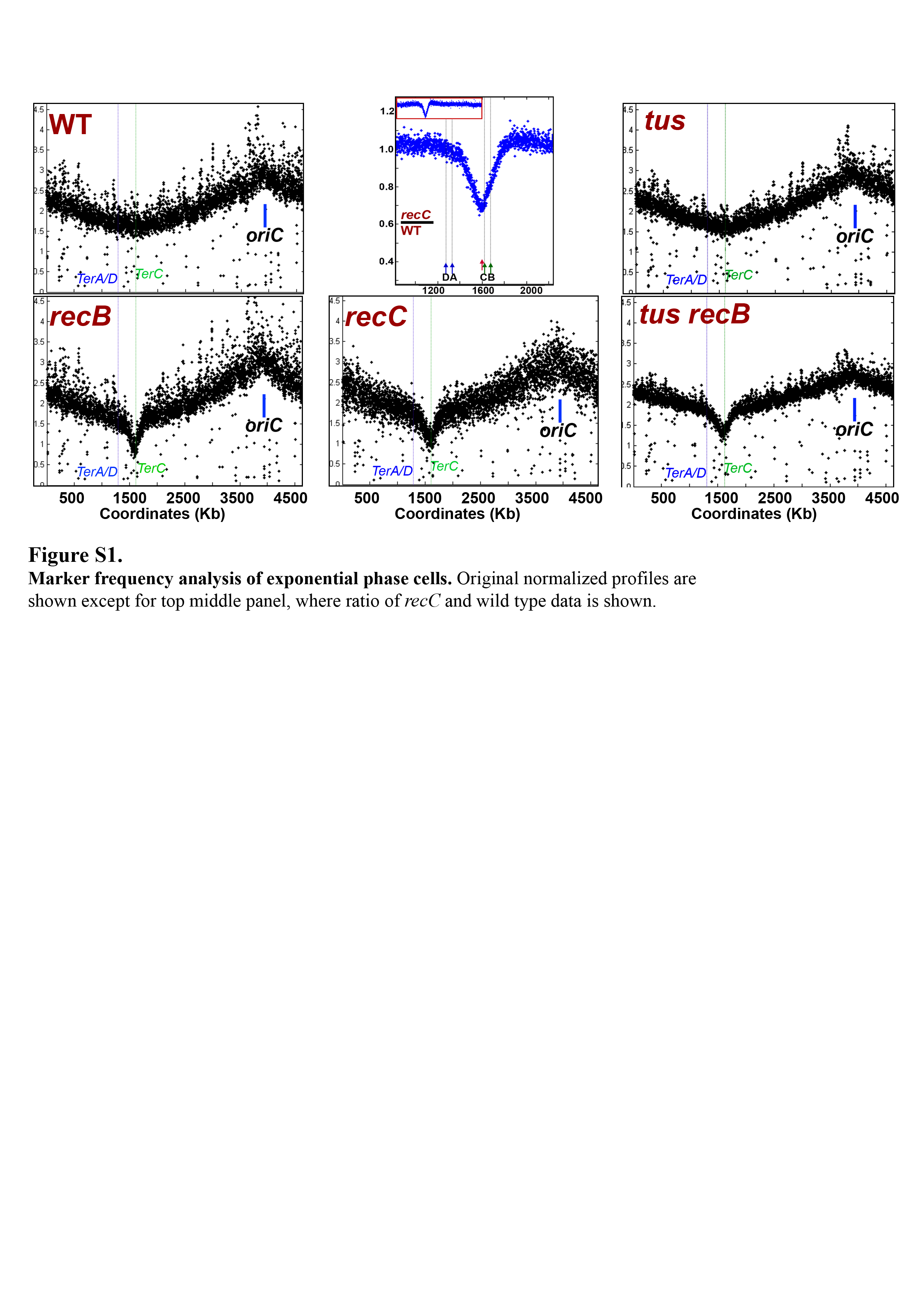

Supplement: S1 Fig — Original normalized profiles are shown except for the top middle panel where ratio of recC and wild-type data is shown. (TIF) [file pgen.1006895.s001.tif]

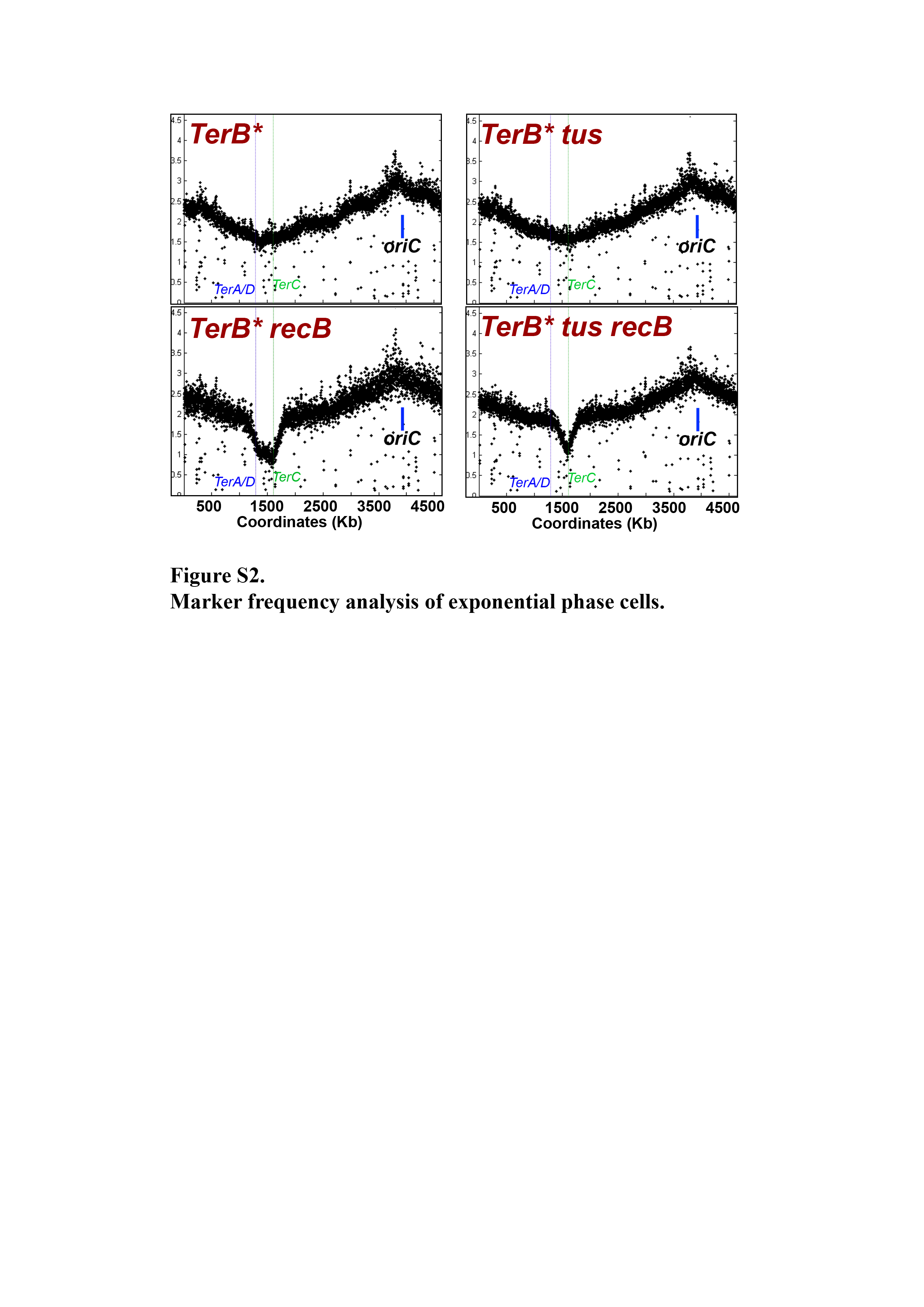

Supplement: S2 Fig — (TIF) [file pgen.1006895.s002.tif]

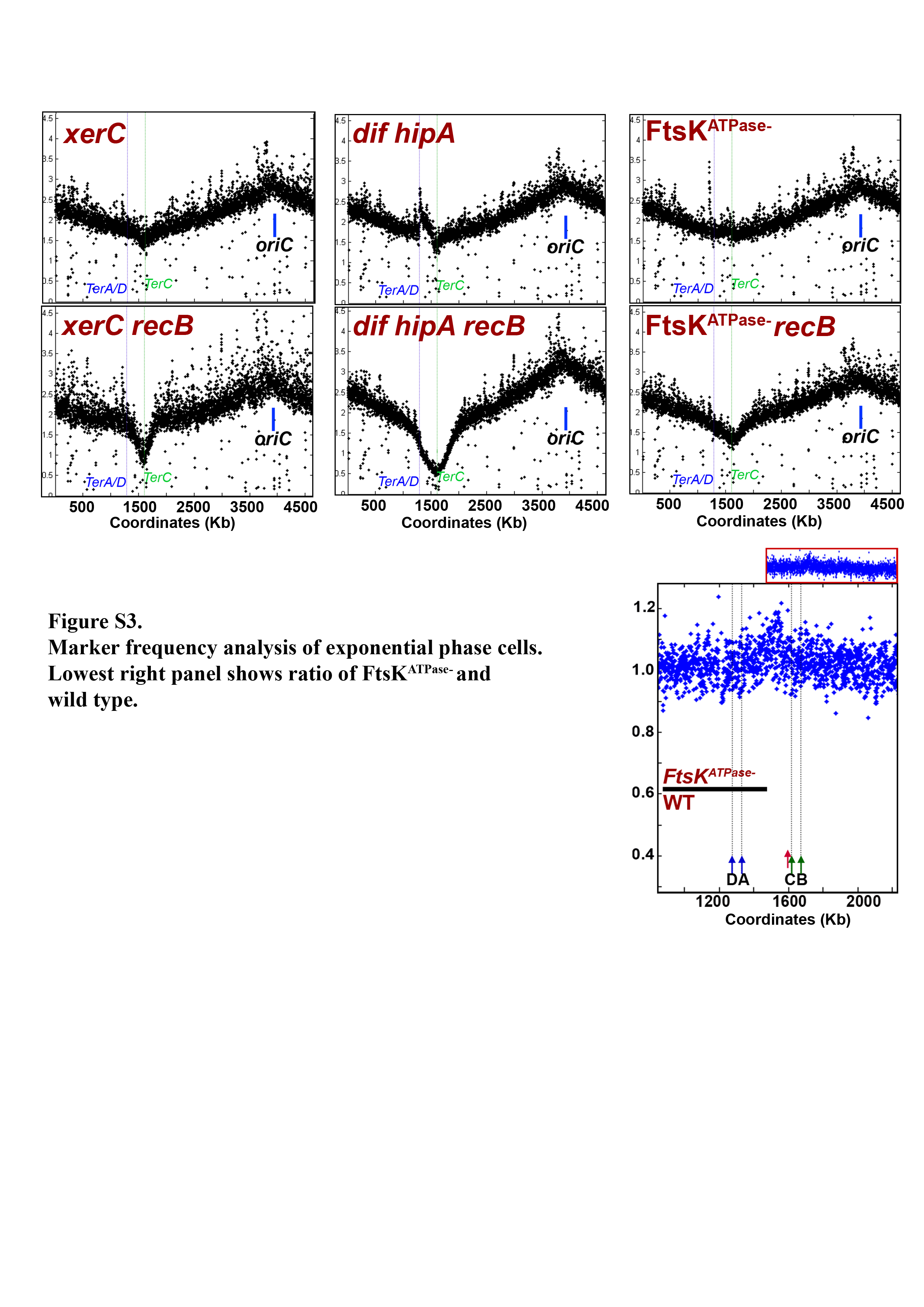

Supplement: S3 Fig — Original normalized profiles are shown except for the bottom panel where ratio of Fts ATPase and wild-type data is shown. (TIF) [file pgen.1006895.s003.tif]

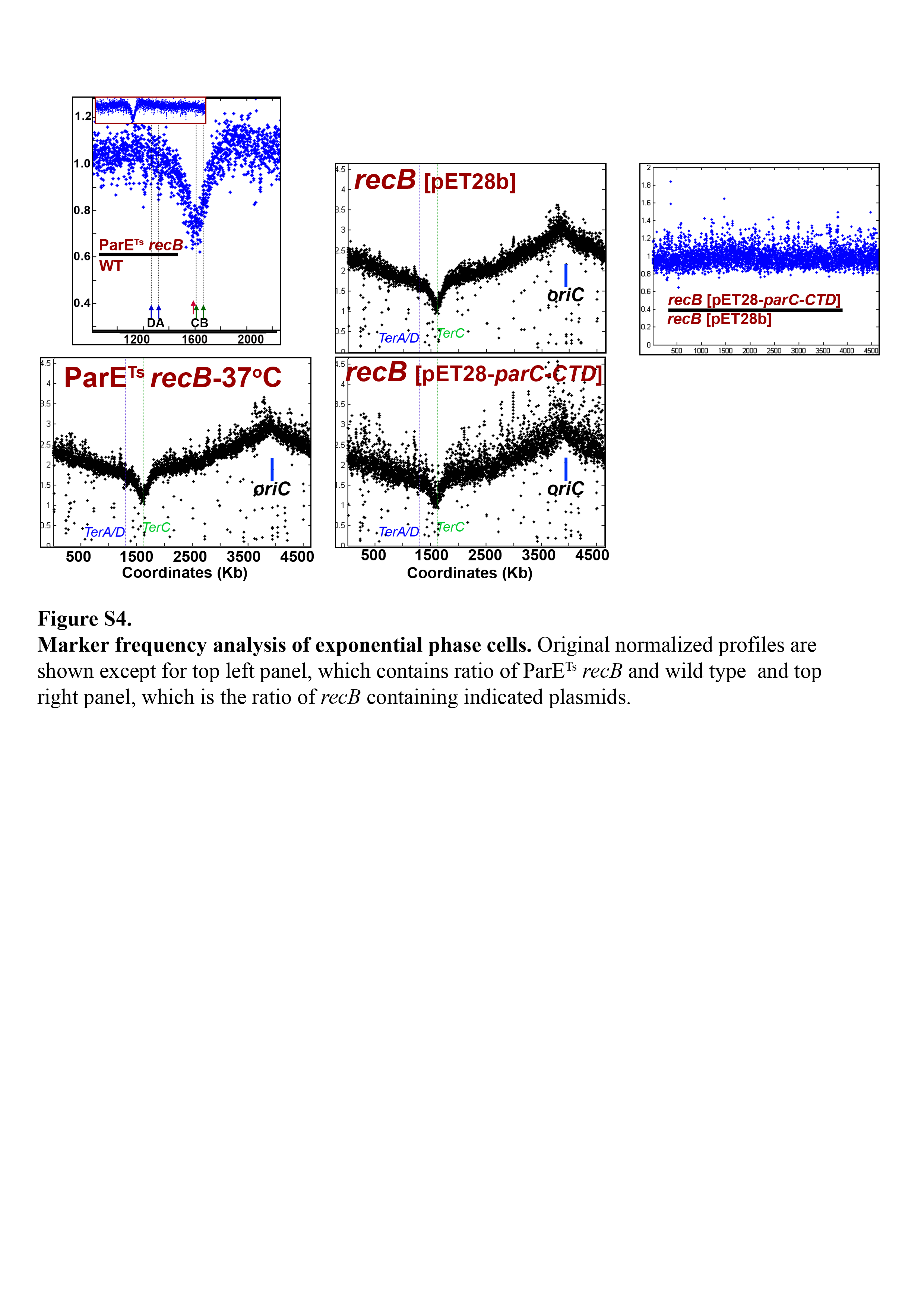

Supplement: S4 Fig — Original normalized profiles are shown except for the top left panel which shows ratio of parEts recB and wild-type, and top right panel which shows the ratio of recB containing the indicated plasmids. (TIF) [file pgen.1006895.s004.tif]

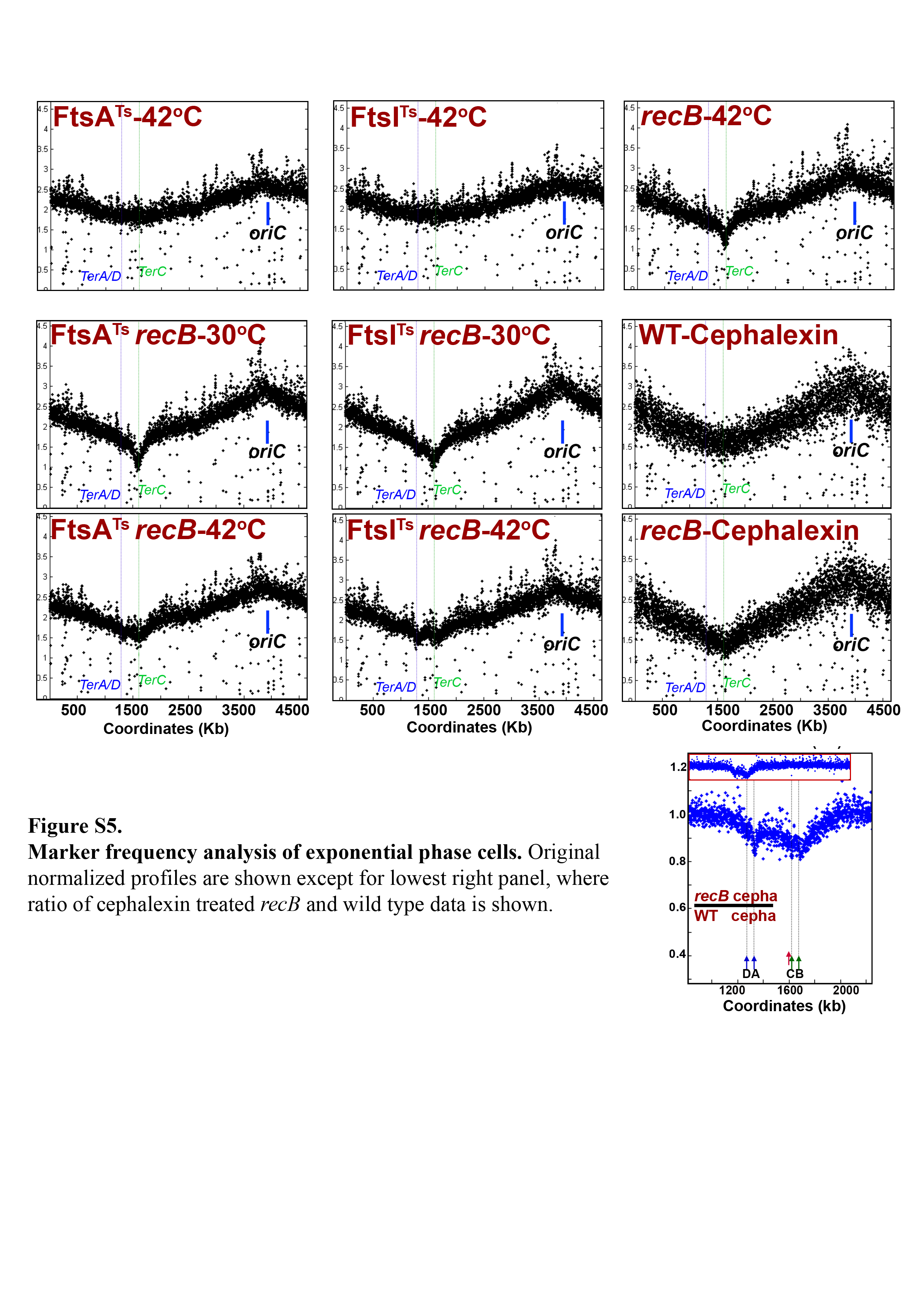

Supplement: S5 Fig — Original normalized profiles are shown except for the lowest right panel where ratio of cephalexin-treated recB and wild-type cells is shown. (TIF) [file pgen.1006895.s005.tif]

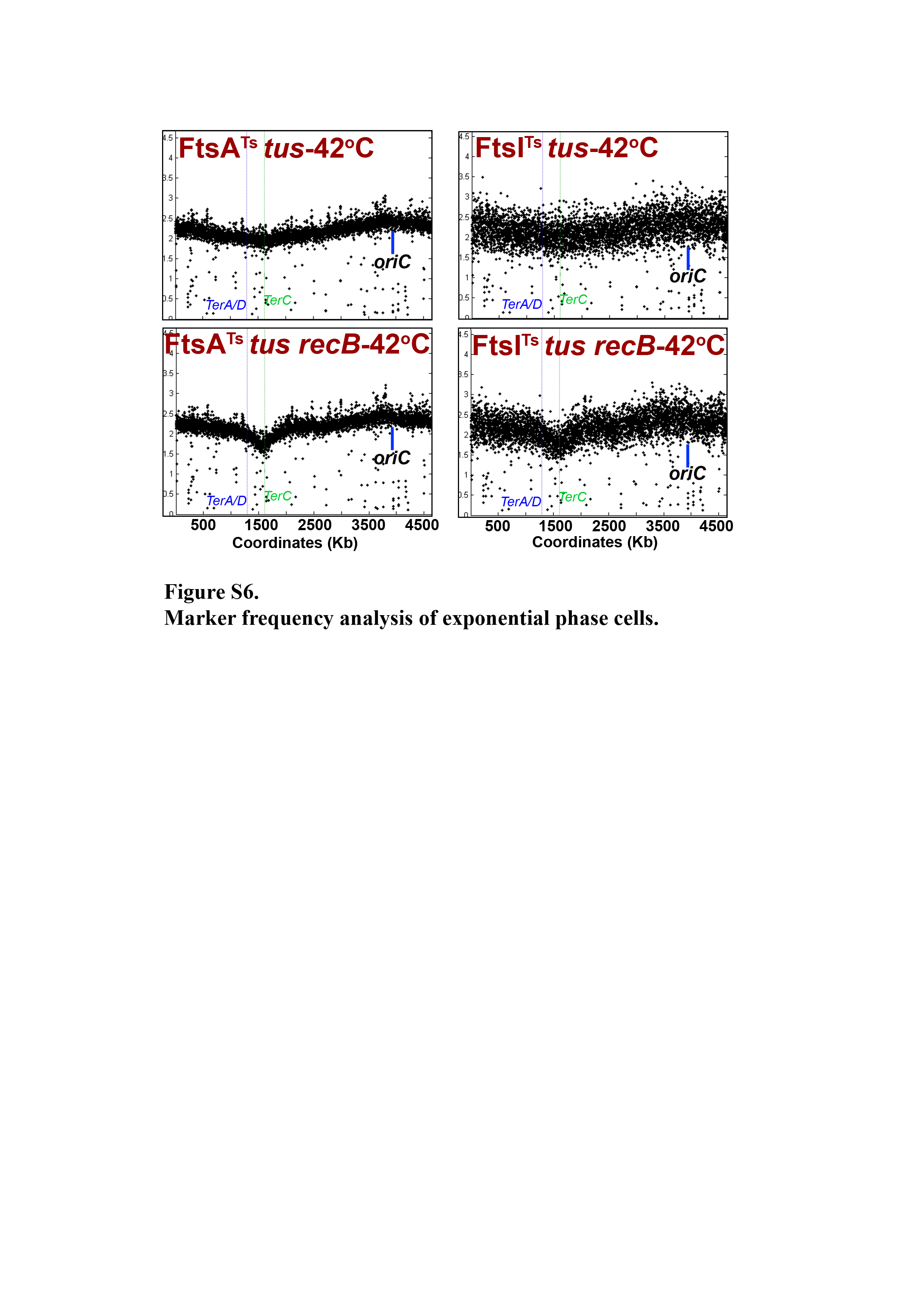

Supplement: S6 Fig — (TIF) [file pgen.1006895.s006.tif]

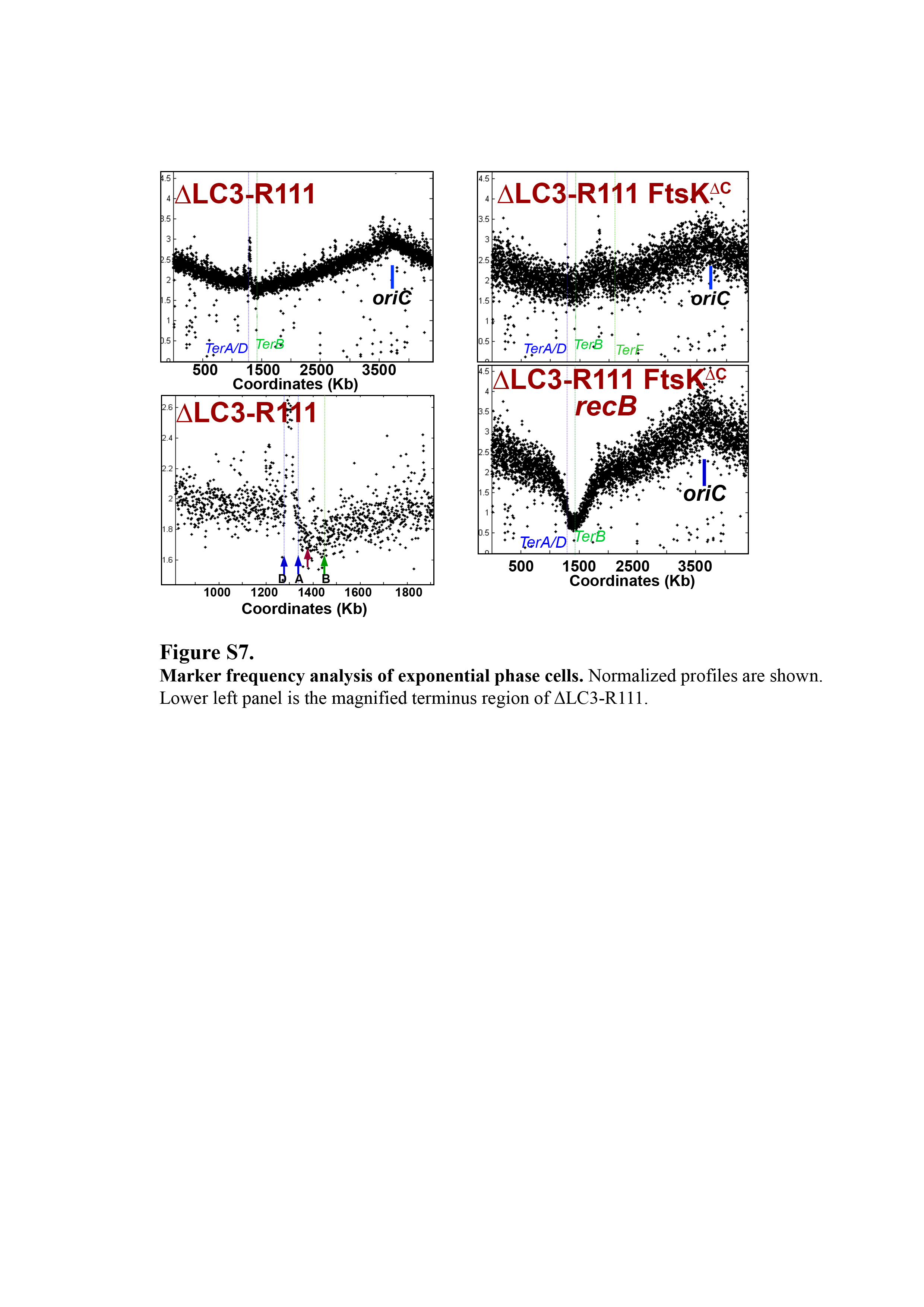

Supplement: S7 Fig — Original normalized profiles are shown. Lower left panel shows the magnified terminus region of ΔLC3-R111 mutant. In ΔLC3-R111 the two replication forks are expected to merge at equal distance from the origin in both directions, between TerA and TerB. However, the MFA shows an excess of reads in the region on the left of TerA on the figure compared to the region on the right, with a breakpoint around TerA, which suggests some DNA amplification left of TerA in a RecB+ context. There is no evidence for this amplification phenomenon in a recB mutant context (Fig 7A) and for this reason we present the results obtained in the ΔLC3-R111 recB mutant and not the ratio of ΔLC3-R111 recB to ΔLC3-R111 RecB+ in Fig 7A. Note that the breakpoint in the number of sequence reads around TerA is not detected in the FtsKΔCTer context, where instead an unexplained amplification is apparent between TerB and TerF. Further work will be needed to fully understand these phenomenon. (TIF) [file pgen.1006895.s007.tif]

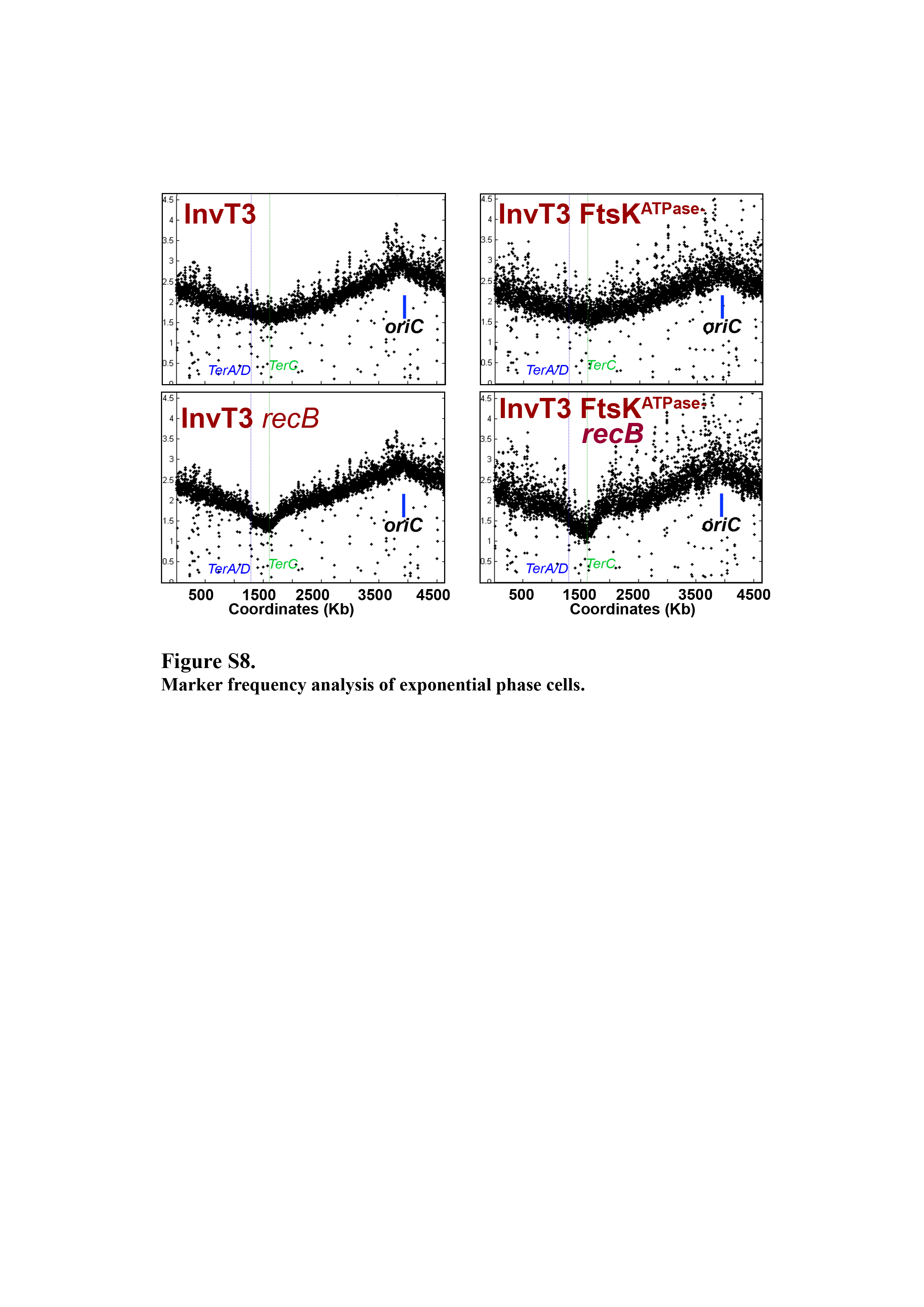

Supplement: S8 Fig — (TIF) [file pgen.1006895.s008.tif]

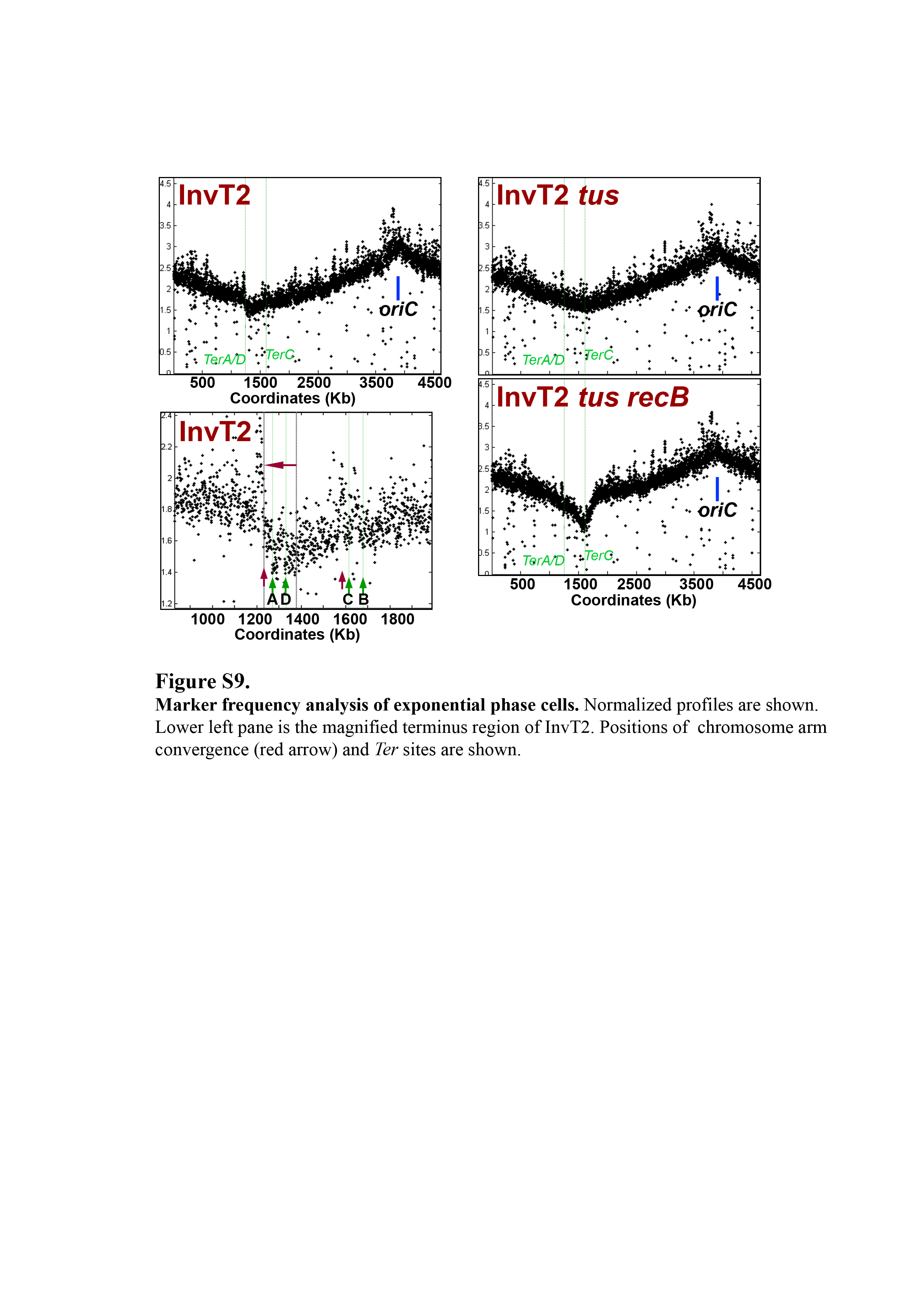

Supplement: S9 Fig — Original normalized profiles are shown. Lower left panel shows the magnified terminus region of InvT2. (TIF) [file pgen.1006895.s009.tif]
