# Supplementary material for: Division-induced DNA double strand breaks in the chromosome terminus region of Escherichia coli lacking RecBCD DNA repair enzyme
Source: PLoS Genet. 2017 Oct 2;13(10):e1006895. doi: 10.1371/journal.pgen.1006895 (PMC5638614; doi:10.1371/journal.pgen.1006895)
Supplement: S1 Table — (PDF) [file pgen.1006895.s010.pdf]

Table S1 Strains

Unless otherwise indicated all strains derive from MG1655

Strain	Relevant genotype	Construction
JJC276	*recD1901*::Tn*10*	Laboratory collection
JJC522	AB1157 Δ*xerC2*::Kan	(Colloms et al., 1990)
JJC777	AB1157 *recB268*::Tn*10* pDWS2	Laboratory collection
JJC1021	AB1157 Δ*dif*::Kan *zie501*::Tn*10*	Laboratory collection
JJC1086	AB1157 Δ*recB*::Kan	Laboratory collection Murphy et al,
JJC1088	AB1157 Δ*dif-hipA*::Ap *sfiA11*	Laboratory collection
JJC1176	*ftsK*$^{\Delta CTer}$ Cm	(Draper et al., 1998)
JJC1377	Δ*recB-recC*::Ap pGB2-RecBCD^{+}	(Corre et al., 1997)
JJC1392	MG1655	Laboratory collection
JJC2264	AB1157 *parE10ts metC*::Tn*10*	(Grompone et al., 2004)
JJC3523	Δ*lacZ*	Laboratory collection
JJC5098	DY330	(Yu et al., 2000)
JJC5235	Δ*tus*::Cm	F. Boccard laboratory collection
JJC5247	Δ*tus*::Cm	JJC1392 * P1 JJC5247
JJC5330	Δ*tus*::FRT	JJC5247 excised of CmR by FRT recombination
JJC5650	*recB268*::Tn*10*	JJC1392 * P1 JJC777
JJC6276	*recC*::Tn*10* [pDWS2]	Laboratory collection

JJC6568	Δ*tus*::Cm Δ*recB*::Kan	JJC5247 * P1 JJC1086
JJC6607	Δ*recB*::Kan	JJC1392 * P1 JJC1086
JJC6618	Δ*xerC2*::Kan	JJC1392 * P1 JJC522
JJC6619	Δ*dif*::Kan	JJC1392 * P1 JJC1021
JJC6621	*parE10ts metC*::Tn*10*	JJC1392 * P1 JJC2264
JJC6628	Δ*xerC2*::Kan *recB268*::Tn*10*	JJC6618 * P1 JJC777
JJC6636	Δ*dif*::Kan *recB268*::Tn*10*	JJC6619* P1 JJC777
JJC6637	*parE10ts metC*::Tn*10* Δ*recB*::Kan	JJC6621 * P1 IIC1086
JJC6640	LC3 (attL 1379 810) R177 (attR 1 229 217) [ptsACXi] CmR KanR	(Valens et al., 2004)
JJC6651	Inv(LC3-R177) = InvT2 CmR KanR	JJC6640 inverted and cured of ptsACXi, Esnault et al, 2007
JJC6652	InvT2 *recB268*::Tn*10*	JJC6652 * P1 JJC777
JJC6719	LC3 (attL 1379 810) R132 (attR 1 554 591) [ptsACXi] CmR KanR	(Esnault et al., 2007)
JJC6727	*ftsK*-Lys997Ala-Cm	FX100 Barre laboratory (*thy* context) ATPase deficient
JJC6728	Inv(LC3-R132) = InvT3 CmR KanR	JJC6719 inverted and cured of ptsACXi (Esnault et al, 2007)
JJC6742	Δ*lacZ ydeV* ::parS$_{pMT1}$ ::Cm	Gift of R. Mercier and O. Espeli, F. Boccard laboratory
JJC6743	Δ*lacZ ydeV* ::parS$_{pMT1}$ ::FRT	F. Boccard laboratory collection
JJC6744	Δ*lacZ yoaC* ::parS$_{pMT1}$ ::FRT	(Espeli et al., 2012)
JJC6755	Δ*lacZ ydeV* ::parS$_{pMT1}$ ::FRT [pHF2973]	JJC6742 transformed by pHF2973
JJC6762	*ftsK*-Lys997Ala-Cm	JJC1392 * P1 JJC6727

JJC6772	*ftsK*-Lys997Ala-Cm *recB268*::Tn*10*	JJC6762 * P1 JJC777
JJC6792	MG58 Δ*lacZ yoaC* ::parS$_{pMT1}$ ::Cm	F. Boccard laboratory collection
JJC6808	Δ*lacZ yoaC* ::parS$_{pMT1}$ ::Cm	JJC3523 * P1 JJC6792
JJC6809	Δ*lacZ ycdN* ::parS$_{pMT1}$ ::Cm	JJC3523 * P1 *ycdN* ::parSpMT1 ::Cm (gift of S. Duigou, F. Boccard laboratory)
JJC6866	DY330 *endA* ::kan	JJC5098 transformed with PCR amplified *endA*::kan
JJC6879	Δ*lacZ ydeV* ::parS$_{pMT1}$ ::FRT [pHF2973] *endA* ::kan	JJC6755 * P1 JJC6866
JJC6900 (a)	Δ*lacZ ydeV* ::parS$_{pMT1}$ ::FRT [pHF2973] *endA* ::kan *recB268*::Tn*10*	JJC6979 * P1 JJC777
JJC6915	*ftsAts leu* ::Tn*10*	F. Boccard laboratory collection
JJC6916	*ftsIts* leu ::Tn*10*	F. Boccard laboratory collection
JJC6973	Δ*lacZ yoaC* ::parS$_{pMT1}$ :: FRT	JJC6808 excised of CmR by FRT recombination
JJC6974	Δ*lacZ ycdN* ::parS$_{pMT1}$ ::FRT	JJC6809 excised of CmR by FRT recombination
JJC6986	Δ*tus*::Tet	F. Boccard laboratory collection
JJC6994	*ftsAts leu* ::Tn*10*	JJC1392 * P1 JJC6915
JJC6995	*ftsIts* leu ::Tn*10*	JJC1392 * JJC6916
JJC6996	InvT2 Δ*tus*::Tet	JJC6651 * P1 JJC6986
JJC7024	*ftsAts leu* ::Tn*10* Δ*recB*::Kan	JJC6994 * P1 JJC1086
JJC7025	*ftsIts* leu ::Tn*10* Δ*recB*::Kan	JJC6995 * P1 JJC1086
JJC7049	InvT2 Δ*tus*::Tet Δ*recB-recC*::Ap	JJC6996 * P1 1377
JJC7056	*ftsK*-Lys997Ala-Cm *zbj1230* ::Tn*10*	JJC6762 * P1 CAG 18478
JJC7064(b)	InvT2 *ftsK*-Lys997Ala-Cm *zbj1230* ::Tn*10*	JJC6651 * P1 JJC7056

JJC7065	InvT3 *ftsK*-Lys997Ala-Cm *zbj1230* ::Tn*10*	JJC6728 * P1 JJC7056
JJC7075	InvT3 *ftsK*-Lys997Ala-Cm *zbj1230* ::Tn*10* Δ*recB-recC*::Ap	JJC6765 * P1 JJC1377
JJC7085	InvT3 *ftsK*-Lys997Ala-Cm *zbj1230* ::Tn*10*	JJC6728 * P1 JJC7056
JJC7121	Δ*tus*::FRT *ftsK*-Lys997Ala-Cm	JJC5330 * P1 JJC6727
JJC7142	Δ*tus*::FRT *ftsK*-Lys997Ala-Cm *recB268*::Tn*10*	JJC7121 * P1 JJC777
JJC7186	*ftsK*-Lys997Ala-Cm Δ*xerC2*::Kan	JJC6762 * P1 JJC522
JJC7190	*ftsK*-Lys997Ala-Cm Δ*xerC2*::Kan *recB268*::Tn*10*	JJC7186 * P1 JJC777
JJC7215	*recB268*::Tn*10* [pET28b]	JJC5650 transformed with pET28b
JJC7216	*recB268*::Tn*10* [pET-ParC-CTD]	JJC5650 transformed with pET- ParC-CTD
JJC7231	*araC* ::*GFP-parB*$_{pMT1}$ ::Cm	JJC5098 transformed with PCR amplified *GFP-parBpMT1* ::Cm
JJC7232	Δ*lacZ ydeV* ::parS$_{pMT1}$ ::FRT *araC* ::*GFP-parB*$_{pMT1}$ ::Cm	JJC6743 * P1 JJC7231
JJC7252	Δ*lacZ ydeV* ::parS$_{pMT1}$ ::FRT *araC* ::*GFP-parB*$_{pMT1}$ ::FRT	JJC7232 excised of CmR by FRT recombination
JJC7259	Δ*lacZ yoaC* ::parS$_{pMT1}$ :: FRT *araC* ::*GFP-parB*$_{pMT1}$ ::Cm	JJC6973 * P1 JJC7231
JJC7260	Δ*lacZ ycdN* ::parS$_{pMT1}$ ::FRT *araC* ::*GFP-parB*$_{pMT1}$ ::Cm	JJC6974 * P1 JJC7231
JJC7261	Δ*lacZ ydeV* ::parS$_{pMT1}$ ::FRT *araC* ::*GFP-parB*$_{pMT1}$ ::FRT *recB268*::Tn*10*	JJC7252 * P1 JJC777
JJC7263	Δ*lacZ ydeV* ::parS$_{pMT1}$ ::FRT *araC* ::*GFP-parB*$_{pMT1}$ ::FRT *xerC2*::Kan	JJC7252 * P1 JJC522
JJC7264	Δ*lacZ ydeV* ::parS$_{pMT1}$ ::FRT *araC* ::*GFP-parB*$_{pMT1}$ ::FRT *ftsK*-Lys997Ala-Cm	JJC7252 * P1 JJC6727
JJC7269	Δ*lacZ yoaC* ::parS$_{pMT1}$ :: FRT *araC* ::*GFP-parB*$_{pMT1}$ ::Cm *recB268*::Tn*10*	JJC7259 * P1 JJC777
JJC7270	Δ*lacZ ycdN* ::parS$_{pMT1}$ ::FRT *araC* ::*GFP-parB*$_{pMT1}$ ::Cm *recB268*::Tn*10*	JJC7260 * P1 JJC777

JJC7271	Δ*lacZ yoaC* ::parS$_{pMT1}$ :: FRT *araC* ::*GFP-parB*$_{pMT1}$ ::Cm *xerC2*::Kan	JJC7259 * P1 JJC522
JJC7272	Δ*lacZ ycdN* ::parS$_{pMT1}$ ::FRT *araC* ::*GFP-parB*$_{pMT1}$ ::Cm *xerC2*::Kan	JJC7260 * P1 JJC522
JJC7273	Δ*lacZ ydeV* ::parS$_{pMT1}$ ::FRT *araC* ::*GFP-parB*$_{pMT1}$ ::FRT *xerC2*::Kan *recB268*::Tn*10*	JJC7263 * P1 JJC777
JJC7274	Δ*lacZ ydeV* ::parS$_{pMT1}$ ::FRT *araC* ::*GFP-parB*$_{pMT1}$ ::FRT *ftsK*-Lys997Ala-Cm *recB268*::Tn*10*	JJC7264 * P1 JJC777
JJC7277	Δ*lacZ yoaC* ::parS$_{pMT1}$ :: FRT *araC* ::*GFP-parB*$_{pMT1}$ ::FRT	JJC7259 excised of CmR by FRT recombination
JJC7278	Δ*lacZ ycdN* ::parS$_{pMT1}$ ::FRT *araC* ::*GFP-parB*$_{pMT1}$ ::FRT	JJC7260 excised of CmR by FRT recombination
JJC7279	Δ*lacZ yoaC* ::parS$_{pMT1}$ :: FRT *araC* ::*GFP-parB*$_{pMT1}$ ::Cm *xerC2*::Kan *recB268*::Tn*10*	JJC7271 * P1 JJC777
JJC7280	Δ*lacZ ycdN* ::parS$_{pMT1}$ ::FRT *araC* ::*GFP-parB*$_{pMT1}$ ::Cm *xerC2*::Kan *recB268*::Tn*10*	JJC7272 * P1 JJC777
JJC7285	Δ*lacZ ydeV* ::parS$_{pMT1}$ ::FRT *araC* ::*GFP-parB*$_{pMT1}$ ::FRT Δ*tus* ::Tet	JJC7252 * P1 JJC6986
JJC7289	Δ*lacZ ydeV* ::parS$_{pMT1}$ ::FRT *araC* ::*GFP-parB*$_{pMT1}$ ::FRT *recD1901*::Tn*10*	JJC7252 * P1 JJC276
JJC7290	Δ*lacZ ydeV* ::parS$_{pMT1}$ ::FRT *araC* ::*GFP-parB*$_{pMT1}$ ::FRT *recB268*::Tn*10* [pET28b]	JJC6761 transformed with pET28b
JJC7291	Δ*lacZ ydeV* ::parS$_{pMT1}$ ::FRT *araC* ::*GFP-parB*$_{pMT1}$ ::FRT *recB268*::Tn*10* [pET-ParC-CTD]	JJC6761 transformed with pET-ParC-CTD
JJC7297	Δ*lacZ ydeV* ::parS$_{pMT1}$ ::FRT *araC* ::*GFP-parB*$_{pMT1}$ ::FRT Δ*tus* ::Tet Δ*recB* ::Kan	JJC7285 * P1 JJC1086
JJC7304	Δ*lacZ yoaC* ::parS$_{pMT1}$ :: FRT *araC* ::*GFP-parB*$_{pMT1}$ ::FRT *ftsK*-Lys997Ala-Cm	JJC7277 * P1 JJC6727
JJC7305	Δ*lacZ yoaC* ::parS$_{pMT1}$ :: FRT *araC* ::*GFP-parB*$_{pMT1}$ ::FRT *recD1901*::Tn*10*	JJC7277 * P1 JJC276
JJC7325	Δ*lacZ ydeV* ::parS$_{pMT1}$ ::FRT *araC* ::*GFP-parB*$_{pMT1}$ ::FRT Δ*dif-hipA*::Ap	JJC7252 * P1 JJC1088
JJC7326	Δ*lacZ yoaC* ::parS$_{pMT1}$ :: FRT *araC* ::*GFP-parB*$_{pMT1}$ ::Cm Δ*dif-hipA*::Ap	JJC7259 * P1 JJC1088

JJC7329	Δ*lacZ ydeV* ::parS$_{pMT1}$ ::FRT *araC* ::*GFP-parB*$_{pMT1}$ ::FRT Δ*dif-hipA*::Ap *recB268*::Tn*10*	JJC7325 * P1 JJC777
JJC7330	Δ*lacZ yoaC* ::parS$_{pMT1}$ :: FRT *araC* ::*GFP-parB*$_{pMT1}$ ::Cm Δ*dif-hipA*::Ap *recB268*::Tn*10*	JJC7326 * P1 JJC777
JJC7334	Δ*lacZ ydeV* ::parS$_{pMT1}$ ::FRT *araC* ::*GFP-parB*$_{pMT1}$ ::FRT *ftsK*-Lys997Ala-Cm Δ*tus* ::Tet	JJC7264 * P1 JJC6986
JJC7337	DY330 *ydeV* ::parS$_{pMT1}$ Apra	Gene replacement of the Cm marker in JJC7321 by the Apra resistance marker
JJC7338	Δ*lacZ ydeV* ::parS$_{pMT1}$ ::FRT *araC* ::*GFP-parB*$_{pMT1}$ ::FRT *ftsK*-Lys997Ala-Cm Δ*tus* ::Tet Δ*recB*::Kan	JJC7334 * P1 JJC1086
JJC7339	InvT2 *ydeV* ::parS$_{pMT1}$::Apra	JJC6651 * P1 JJC7337
JJC7340	InvT3 *ydeV* ::parS$_{pMT1}$::Apra	JJC6728 * P1 JJC7337
JJC7345	Δ*dif*::Tet	F.X. Barre laboratory collection
JJC7357	Δ*lacZ ydeV* ::parS$_{pMT1}$ ::FRT *araC* ::*GFP-parB*$_{pMT1}$ ::FRT *ftsK*$^{\Delta CTer}$ Cm	JJC7252 * P1 JJC1176
JJC7361	Δ*lacZ ydeV* ::parS$_{pMT1}$ ::FRT *araC* ::*GFP-parB*$_{pMT1}$ ::Cm Δ*dif*::Tet	JJC7252 * P1 JJC7345
JJC7364	Δ*lacZ ydeV* ::parS$_{pMT1}$ ::FRT *araC* ::*GFP-parB*$_{pMT1}$ ::FRT *ftsK*$^{\Delta CTer}$ Cm *recB268*::Tn*10*	JJC7357 P1 JJC777
JJC7366	Δ*lacZ ydeV* ::parS$_{pMT1}$ ::FRT *araC* ::*GFP-parB*$_{pMT1}$ ::Cm Δ*dif*::Tet Δ*recB*::Kan	JJC7361 * P1 JJC1086
JJC7369	DY330 *araC* ::*GFP-parB*$_{pMT1}$ ::Apra	Gene replacement of the Cm marker in JJC7231 by the Apra resistance marker
JJC7371	Δ*lacZ yoaC* ::parS$_{pMT1}$ :: FRT *araC* ::*GFP-parB*$_{pMT1}$ ::FRT *ftsK*$^{\Delta CTer}$ Cm	JJC7277 * P1 JJC1176
JJC7373	InvT2 *ydeV* ::parS$_{pMT1}$::FRT	JJC7339 excised of Apra by FRT recombination
JJC7374	InvT3 *ydeV* ::parS$_{pMT1}$::FRT	JJC7341 excised of Apra by FRT recombination
JJC7383	InvT2 *ydeV* ::parS$_{pMT1}$::FRT *araC* ::*GFP-parB*$_{pMT1}$ ::Apra	JJC7373 * P1 JJC7369
JJC7384	InvT3 *ydeV* ::parS$_{pMT1}$::FRT *araC* ::*GFP-parB*$_{pMT1}$ ::Apra	JJC7374 * P1 JJC7369

JJC7387	InvT2 *ydeV* ::parS$_{pMT1}$::FRT *araC* ::*GFP-parB*$_{pMT1}$ ::Apra *recB268*::Tn*10*	JJC7383 * P1 JJC777
JJC7388	InvT3 *ydeV* ::parS$_{pMT1}$::FRT *araC* ::*GFP-parB*$_{pMT1}$ ::Apra *recB268*::Tn*10*	JJC7384 * P1 JJC777
JJC7393	DY330 Δ*tus* ::Tet	JJC5098 * P1 JJC6986
JJC7397	Δ*lacZ ycdN* ::parS$_{pMT1}$ ::FRT *araC* ::*GFP-parB*$_{pMT1}$ ::Cm *recD1901*::Tn*10*	JJC7260 * P1 JJC276
JJC7399	Δ*lacZ yoaC* ::parS$_{pMT1}$ :: FRT *araC* ::*GFP-parB*$_{pMT1}$ ::Cm Δ*tus* ::Tet	JJC7259 * P1 JJC6986
JJC7401	DY330 Δ*tus* ::Tet *pspE*::*TerB::*Cm	Gene replacement of the *pspE* gene by a PCR fragment carrying *TerB* and the CmR marker
JJC7405	Δ*lacZ yoaC* ::parS$_{pMT1}$ :: FRT *araC* ::*GFP-parB*$_{pMT1}$ ::FRT *ftsK*$^{\Delta CTer}$ Cm *recB268*::Tn*10*	JJC7371 * P1 JJC777
JJC7406	Δ*lacZ ydeV* ::parS$_{pMT1}$ ::FRT *araC* ::*GFP-parB*$_{pMT1}$ ::FRT *pspE*::*Ter::*Cm	JJC7252 * P1 JJC7401
JJC7407	Δ*lacZ ydeV* ::parS$_{pMT1}$ ::FRT *araC* ::*GFP-parB*$_{pMT1}$ ::FRT Δ*tus* ::Tet *pspE*::*TerB::*Cm	JJC7285 * P1 JJC7401
JJC7408	Δ*lacZ yoaC* ::parS$_{pMT1}$ :: FRT *araC* ::*GFP-parB*$_{pMT1}$ ::Cm Δ*tus* ::Tet Δ*recB*::Kan	JJC7399 * P1 JJC1086
JJC7411	Δ*lacZ yoaC* ::parS$_{pMT1}$ :: FRT *araC* ::*GFP-parB*$_{pMT1}$ ::FRT *ftsK*-Lys997Ala-Cm *recB268*::Tn*10*	JJC7304 * P1 JJC777
JJC7415	Δ*lacZ ydeV* ::parS$_{pMT1}$ ::FRT *araC* ::*GFP-parB*$_{pMT1}$ ::FRT *pspE*::*Ter::*Cm *recB268*::Tn*10*	JJC7406 * P1 JJC777
JJC7422	Δ*lacZ ydeV* ::parS$_{pMT1}$ ::FRT *araC* ::*GFP-parB*$_{pMT1}$ ::FRT Δ*tus* ::Tet *pspE*::*TerB::*Cm Δ*recB*::Kan	JJC7407 * P1 JJC1086
JJC7431	*pspE*::*TerB::*Cm	JJC1392 * P1 JJC7401
JJC7432	Δ*lacZ yoaC* ::parS$_{pMT1}$ :: FRT *araC* ::*GFP-parB*$_{pMT1}$ ::FRT *pspE*::*TerB::*Cm	JJC7277 * P1 JJC7401
JJC7433	Δ*lacZ ycdN* ::parS$_{pMT1}$ ::FRT *araC* ::*GFP-parB*$_{pMT1}$ ::FRT *pspE*::*TerB::*Cm	JJC7278 * P1 7401
JJC7435	*pspE*::*TerB::*Cm *recB268*::Tn*10*	JJC7431 * P1 JJC777

JJC7436	Δ*lacZ yoaC* ::parS$_{pMT1}$ :: FRT *araC* ::*GFP-parB*$_{pMT1}$ ::FRT *pspE*::*TerB::*Cm *recB268*::Tn*10*	JJC7432 * P1 JJC777
JJC7437	Δ*lacZ ycdN* ::parS$_{pMT1}$ ::FRT *araC* ::*GFP-parB*$_{pMT1}$ ::FRT *pspE*::*TerB::*Cm *recB268*::Tn*10*	JJC33 * P1 JJC777
JJC7438	*pspE*::*TerB::*Cm Δ*tus*::Tet	JJC7431 * P1 JJC6986
JJC7440	LC3 (AttL-Cm 1 379 810) R111 (AttR Kan 1 617 226)	(Valens et al., 2004)
JJC7443	*pspE*::*TerB::*Cm Δ*tus*::Tet Δ*recB*::Kan	JJC7438 * P1 1086
JJC7444	Δ*lacZ ydeV* ::parS$_{pMT1}$ ::FRT *araC* ::*GFP-parB*$_{pMT1}$ ::FRT *pspE*::*Ter::*FRT	JJC7406 excised of CmR by FRT recombination
JJC7445	ΔLC3-R111	Excision of the sequence from attL 1 379 810 to attR 1 617 226 by lambda recombination. CmS, KanS ApS (plasmid cured) lacZ+
JJC7448	*ftsAts leu* ::Tn*10* Δ*tus*::Cm	JJC6994 * P1 JJC5247
JJC7449	*ftsIts* leu ::Tn*10* Δ*tus*::Cm	JJC6995 * P1 JJC5247
JJC7452	ΔLC3-R111 *araC* ::*GFP-parB*$_{pMT1}$ ::Apra	JJC7445 * P1 JJC7369
JJC7457	DY330 *pspE*:: *parS* $_{pMT1}$*::*Cm	Gene replacement of the *pspE* gene by a PCR fragment carrying *parS* $_{pMT1}$ and the CmR marker
JJC7458	ΔLC3-R111 *araC* ::*GFP-parB*$_{pMT1}$ ::Apra Δ*lacZ yoaC* ::parS$_{pMT1}$ ::Cm	JJC7458 * P1 JJC6792
JJC7459	ΔLC3-R111 *araC* ::*GFP-parB*$_{pMT1}$ ::Apra *pspE*:: *parS* $_{pMT1}$*::*Cm	JJC7452 * P1 JJC7457
JJC7461(c)	Δ*lacZ ydeV* ::parS$_{pMT1}$ ::FRT *araC* ::*GFP-parB*$_{pMT1}$ ::FRT *pspE*::*Ter::*FRT *ftsK*$^{\Delta CTer}$ Cm	JJC7444 * P1 JJC1176
JJC7477	*ftsAts leu* ::Tn*10* Δ*tus*::Cm Δ*recB*::Kan	JJC7448 * P1 JJC1086
JJC7478	*ftsIts* leu ::Tn*10* Δ*tus*::Cm Δ*recB*::Kan	JJC7449 * P1 JJC1086
JJC7485	ΔLC3-R111 *araC* ::*GFP-parB*$_{pMT1}$ ::Apra *pspE*:: *parS* $_{pMT1}$*::*Cm *recB268*::Tn*10*	JJC7459 * P1 JJC777

JJC7497	ΔLC3-R111 *recB268*::Tn*10*	JJC7445 * P1 JJC777
JJC7503	ΔLC3-R111 *ftsK*$^{\Delta CTer}$ Cm	JJC7445 * P1 JJC1176
JJC7504	ΔLC3-R111 *araC* ::*GFP-parB*$_{pMT1}$ ::Apra Δ*lacZ yoaC* ::parS$_{pMT1}$ ::Cm [pAM-RecBCD]	JJC7458 * pAM-RecBCD
JJC7509	ΔLC3-R111 *ftsK*$^{\Delta CTer}$ Cm *recB268*::Tn*10*	JJC7503 * P1 JJC777
JJC7510(d)	ΔLC3-R111 *araC* ::*GFP-parB*$_{pMT1}$ ::Apra Δ*lacZ yoaC* ::parS$_{pMT1}$ ::Cm [pAM-RecBCD] *recB268*::Tn*10*	JJC7504 * P1 JJC777
JJC7528	Δ*lacZ ydeV* ::parS$_{pMT1}$ ::FRT *araC* ::*GFP-parB*$_{pMT1}$ ::FRT *recC*::Tn*10*	JJC7252 * P1 JJC6276
JJC7529	Δ*lacZ yoaC* ::parS$_{pMT1}$ :: FRT *araC* ::*GFP-parB*$_{pMT1}$ ::FRT *recC*::Tn*10*	JJC7277 * P1 JJC6276
JJC7530	*recC*::Tn*10*	JJC1392 * P1 JJC6276

(a) In 6879 and 6900, ParB $_{pMT1}$ is expressed from pHF2973 (Nielsen et al., 2006).

(b) JJC7064 produced colonies of very heterogeneous sizes when streaked on LB as on M9 plates. This poorly growing mutant could therefore not be used.

(c) JJC7461 was transduced twice with a *recB268*::Tn*10* P1 stock, yielding 4 and 6 little colonies in three days, respectively. These colonies could not be streaked while P1 transduction of other strains realized in parallel produced the expected number of clones with the expected size and phenotype. We conclude that the *pspE*::*TerB ftsK*$^{\Delta CTer}$ *recB* combination of mutations is nearly lethal and cannot be tested.

(d) pAM-RecBCD^{+} plasmid is a plasmid that carries the entire *recB recC recD* region and replicates only in the presence of IPTG. JJC7510 was constructed in its presence because of the slow growth of the ΔLC3-R111 *recB268*::Tn*10* mutant. The plasmid was cured from JJC7510 prior to each experiment by growing cells in the absence of IPTG.

References

Colloms, S.D., Sykora, P., Szatmari, G. and Sherratt, D.J. (1990) Recombination at ColE1 cer requires the Escherichia coli xerC gene product, a member of the lambda-integrase family of site-specific recombinases. *J Bacteriol*, **172**, 6973-6980.

Corre, J., Cornet, F., Patte, J. and Louarn, J.M. (1997) Unraveling a region-specific hyper-recombination phenomenon: Genetic control and modalities of terminal recombination in Escherichia coli. *Genetics*, **147**, 979-989.

Cui, T., Moro-oka, N., Ohsumi, K., Kodama, K., Ohshima, T., Ogasawara, N., Mori, H., Wanner, B., Niki, H. and Horiuchi, T. (2007) Escherichia coli with a linear genome. *EMBO Rep*, **8**, 181-187.

Draper, G.C., McLennan, N., Begg, K., Masters, M. and Donachie, W.D. (1998) Only the N-terminal domain of FtsK functions in cell division. *J Bacteriol*, **180**, 4621-4627.

Esnault, E., Valens, M., Espeli, O. and Boccard, F. (2007) Chromosome structuring limits genome plasticity in Escherichia coli. *PLoS Genet*, **3**, e226.

Espeli, O., Borne, R., Dupaigne, P., Thiel, A., Gigant, E., Mercier, R. and Boccard, F. (2012) A MatP-divisome interaction coordinates chromosome segregation with cell division in E. coli. *Embo J*, **31**, 3198-3211.

Grompone, G., Bidnenko, V., Ehrlich, S.D. and Michel, B. (2004) PriA is essential for viability of the Escherichia coli topoisomerase IV parE10(Ts) mutant. *J Bacteriol*, **186**, 1197-1199.

Nielsen, H.J., Ottesen, J.R., Youngren, B., Austin, S.J. and Hansen, F.G. (2006) The Escherichia coli chromosome is organized with the left and right chromosome arms in separate cell halves. *Mol Microbiol*, **62**, 331-338.

Valens, M., Penaud, S., Rossignol, M., Cornet, F. and Boccard, F. (2004) Macrodomain organization of the Escherichia coli chromosome. *Embo J*, **23**, 4330-4341.

Yu, D., Ellis, H.M., Lee, E.C., Jenkins, N.A., Copeland, N.G. and Court, D.L. (2000) An efficient recombination system for chromosome engineering in Escherichia coli. *Proc Natl Acad Sci U S A*, **97**, 5978-5983.
