# Supplementary material for: Division-induced DNA double strand breaks in the chromosome terminus region of Escherichia coli lacking RecBCD DNA repair enzyme
Source: PLoS Genet. 2017 Oct 2;13(10):e1006895. doi: 10.1371/journal.pgen.1006895 (PMC5638614; doi:10.1371/journal.pgen.1006895)
Supplement: S2 Table — (PDF) [file pgen.1006895.s011.pdf]

S2 Table. Percentage of cells with zero, one or two foci in mutant strains.

ydeV::parS $_{\mathbf{pMT1}}$	% of cells				N (n)
Foci per cells	0	1	2	>2	
wt	0.6 ± 0.2	81.2 ± 2.1	18.1 ± 2.3	0.12 ± 0.1	1547 (3)
recB	32 ± 1.5	57.2 ± 2.2	10.1 ± 1.9	0.7 ± 0.15	1397 (3)
recC	30.6 ± 1.6	59.2 ± 3.9	8.7 ± 4.9	1.5 ± 0.7	994 (2)
tus	0.8 ± 0.24	82.8 ± 1.7	16.2 ± 2.1	0.16 ± 0.2	633 (2)
tus recB	36.7 ± 2.1	53.4 ± 0.1	9 ± 1.5	0.95 ± 0.6	1169 (2)
pspE::TerB	0.3 ± 0.3	78.9 ± 2.8	20.5 ± 3.3	0.2 ± 0.2	1090 (3)
pspE::TerB recB	48 ± 7	41.2 ± 3.2	9.3 ± 3.2	1.5 ± 1.3	1256 (3)
pspE::TerB tus	0.8 ± 0.06	84.7 ± 2.5	14.5 ± 2.4	0.07 ± 0.1	1441 (3)
pspE::TerB tus recB	35.1 ± 5.6	55.1 ± 9.5	9.5 ± 3.8	0.4 ± 0.4	1240 (3)
xerC	14.8 ± 1	69.7 ± 2	14.7 ± 0.5	0.76 ± 0.6	1057 (3)
xerC recB	40.4 ± 0.9	51.9 ± 1.9	7.3 ± 1	0.6 ± 0.44	983 (3)
dif	15.2 ± 1.8	65.4 ± 1.9	19 ± 0.4	0.4 ± 0.3	2020 (3)
dif recB	41.5 ± 2	47.3 ± 2	10.8 ± 1.1	0.47 ± 0.3	1744 (3)
dif hipA	15.7 ± 2.5	71.2 ± 6	11.3 ± 2.8	1.8 ± 0.7	717 (2)
dif hipA recB	64.6 ± 7	31 ± 4.8	3.7 ± 2	0.7 ± 0.6	1228 (3)
ftsK^{ATPase}	20.6 ± 0.4	58.5 ± 1.9	18.2 ± 1.1	2.6 ± 0.3	1387 (2)
ftsK^{ATPase} *recB*	54.7 ± 0.1	33.6 ± 0.1	8.5 ± 0.5	3.2 ± 0.4	1109 (2)
ftsKΔCter	25.1 ± 1.9	53.4 ± 2.1	17.1 ± 2.7	4.3 ± 1.4	1298 (3)
ftsKΔCter recB	54.4 ± 1.2	32.9 ± 2.5	11.9 ± 2.1	1.1 ± 0.3	1273 (3)
ftsK^{ATPase} *tus*	27.3 ± 0.7	67 ± 3.3	13.6 ± 2.8	19 ± 1.6	1534 (3)
ftsK^{ATPase} *tus recB*	49.8 ± 1.2	36.9 ± 1.7	11.9 ± 1	1.4 ± 0.8	1472 (3)
recB [pET28]	34.2 ± 2.4	55.9 ± 0.5	9.4 ± 1.7	0.55 ± 0.25	1089 (3)
recB [pET-parC-TD]	32.4 ± 4.7	57 ± 2.3	10.3 ± 2.7	0.4 ± 0.4	1101 (3)
endA recB[a]	35.2 ± 3.7	54.3 ± 4.7	9.6 ± 1.8	0.4 ± 0.14	775 (2)
InvT3	4.9 ± 2.1	78.5 ± 2.6	15.8 ± 3	1.1 ± 0.9	1158 (3)
InvT3 *recB*	38.4 ± 3.6	52.6 ± 3.5	7.7 ± 1.8	1.2 ± 1.1	1156 (3)
InvT2	8.7 ± 3.7	72.7 ± 1.4	16.6 ± 3.6	1.9 ± 2	1011 (3)

InvT2 *recB*	43.5 ± 3.4	42.5 ± 4.6	9.5 ± 1.7	4.6 ± 1.7	1279 (3)

***pspE*::*parS*$_{\mathbf{pMT1}}$**	% of cells				N
Foci per cells	0	1	2	>2	
recB	15.6 ± 2.7	51.4 ± 5.1	31.8 ± 6.3	1.2 ± 1.4	986 (2)
ΔLC3-R111	16.6 ± 1.9	60.7 ± 2.7	22 ± 2.7	0.8 ± 0.9	1108 (3)
ΔLC3-R111 *recB*	49.2 ± 2.5	40.4 ± 0.5	10.1 ± 1.9	0.24 ± 0.2	1174 (2)
ΔLC3-R111 *tus*	18.4 ± 1.4±	55.1 ± 4.3	23.8 ± 6.2	2.6 ± 0.4	1011 (2)
ΔLC3-R111 *recB tus*	60.5 ± 3.5	27.2 ± 3	9.8 ± 1.5	1.9 ± 2.7	844 (2)

***yoaC*::*parS*$_{\mathbf{pMT1}}$**	% of cells				N (n)
Foci per cells	0	1	2	>2	
wt	0.6 ± 0.3	58.4 ± 1.1	40.7 ± 1.6	0.2 ± 0.19	1491 (3)
recB	7.9 ± 1	51.3 ± 1.1	38 ± 1.4	2.8 ± 0.9	1420 (3)
recC	7.8 ± 1.6	56.7 ± 14.6	33.9 ± ?	1.64 ± 1.4	624 (2)
tus	2.5 ± 0.3	75.4 ± 2.5	22.1 ± 2.9	0 ± 0	917 (2)
tus recB	13.1 ± 0.8	53.3 ± 5.3	30.9 ± 5.5	2.8 ± 0.6	854 (2)
pspE::TerB recB	8.8 ± 7	54.9 ± 1.8	34.9 ± 3	1.45 ± 0.6	1093 (3)
xerC	1.7 ± 1.4	55.2 ± 1.5	41.1 ± 0.3	2 ± 0.4	552 (2)
xerC recB	14 ± 2.6	53.7 ± 1.7	30.3 ± 3.9	2 ± 1.3	1037 (3)
dif hipA	3.6	55.4	39	2.2	359 (1)
dif hipA recB	40 ± 3.2	30 ± 1.9	25 ± 0.3	4.8 ± 1.3	920 (2)
ftsK-ATPase	5.9 ± 0.6	59.8 ± 2.6	28.5 ± 0.3	5.7 ± 1.6	821 (2)
ftsK-ATPase recB	14 ± 0.08	55.7 ± 1.1	26.3 ± 2.6	4.1 ± 1.4	552 (2)
ftsKΔCter	4.5 ± 2.3	42.4 ± 3.5	47.2 ± 5.4	5.9 ± 1.4	1056 (3)
ftsKΔCter recB	15.9 ± 3.1	41.3 ± 5.4	36.1 ± 2.2	6.7 ± 1.6	1245 (3)
ΔLC3-R111 *recB*	10.4 ± 0.06	49.6 ± 3	34.1 ± 5.3	5.8 ± 2.3	1102 (2)

***ycdN*::*parS*$_{\mathbf{pMT1}}$**	% of cells				N
Foci per cells	0	1	2	>2	

wt	0.95 ± 0.88	59.2 ± 3.2	39.7 ± 3.9	0.17 ± 0.2	684 (2)
recB	6.7 ± 0.17	55.5 ± 1.1	36.2 ± 0	1.6 ± 0.95	625 (2)
pspE::TerB recB	7.6 ± 0.7	43.1 ± 4.6	45.5 ± 3.7	3.9 ± 1.7	1178 (3)
xerC	1.8 ± 0.3	48.5 ± 0.1	45.6 ± 1.3	4.1 ± 0.9	660 (2)
xerC recB	5.2 ± 0.07	50.9 ± 3.6	41.5 ± 1.9	2.4 ± 1.8	591 (2)

N = number of cells analyzed. (n) number of independent experiments
IE = proportion of initial events, i.e. of divisions that lead to one daughter cell that shows a focus and one daughter cell with no focus, not counting the divisions where the event is transmitted to progeny.
(a) tested with a plasmid-born *parB*-GFP gene
