# Supplementary material for: Division-induced DNA double strand breaks in the chromosome terminus region of Escherichia coli lacking RecBCD DNA repair enzyme
Source: PLoS Genet. 2017 Oct 2;13(10):e1006895. doi: 10.1371/journal.pgen.1006895 (PMC5638614; doi:10.1371/journal.pgen.1006895)
Supplement: S3 Table — (PDF) [file pgen.1006895.s012.pdf]

S3 Table- List of the oligonucleotides used in this study

**List of the oligonucleotides used for strain construction**		
532<br>533	AATTCTCGCTGATGTCTCGG<br>GCATCCATTAGTGTCTTTAGG	Primers to verify *ydeV*::$parS_{\mathrm{pMT1}}$
534<br>535	AGCCCTGGATTTCATTGGTG<br>ACCTTCACTCACCCATGAAC	Primers to verify *yoaC*::$parS_{\mathrm{pMT1}}$
592<br><br>593	CGATGTTCGTGTTCCAGAGCAGTATCAGCAAGAGCACGTTCAG GGGGCCAGTGTAGGCTGGAGCTGCTTC<br>CGGAACGGCGGTGGCAATGCGCTCTTTCACTTCCTTCAGGGGA ATATTGACTTACCCGTCTTACTGTCGG	Primers to insert $parS_{\mathrm{pMT1}}$ in the *pspE* gene (Same primers were used to insert *TerB* in *pspE*)
594<br>595	ATCCATAGAAGGACGCTTAC<br>TTAACCTTTGACCTTCGGCA	Primers to verify *pspE*::$parS_{\mathrm{pMT1}}$ and *pspE*::*TerB*
568<br>569	GACTTCAAGCTTTGTGTAGGCTGGAGCTGCTT<br>GACTTCAAGCTTCATATGAATATCCTCCTTAG	Primers with HindIII site was used to amplify cm^R gene from pKD3 plasmid
582<br><br>583	AATATGGACAATTGGTTTCTTCTCTGAATGGTGGGAGTATGAA AAGTATGTCTAAAGGTGAAGAACTGTT<br>ACAGGTCGCTGAAATGCGGCTGGTGCGCTTCATCCGGGCGAAA GAAACCCTGAATATCCTCCTTAGTTCC	Primers to replace *araC* gene with *yGFP*-$parB_{\mathrm{pMT1}}$- cm^R fragment
581<br>597	GCAGCTTCGCCTGACTAATG<br>GACGCAGTGACGGCAATGTC	Primers to verify *araC*:: *yGFP*-$parB_{\mathrm{pMT1}}$- cm^R
584<br><br>585	ATGGAGAAAAAAATCACTGGATATACCACCGTTGATATATCCC AATGGCAATCGCGCGCGCTTCGTTCGGGACGAA<br>TCATCGCAGTACTGTTGTATTCATTAAGCATCTGCCGACATGG AAGCCATATCTGCCTCTTCGTCCCGAAGCAACT	Primers to replace cm^R gene with $apra^R$ gene
537<br><br>538	CTGATCTGGCTGATTGCATACCAAAACAGCTTTCGCTACGTTG CTGGCTCGTGTAGGCTGGAGCTGCTTC<br>TTCGCGCCTGGCAAGCGCGTTGCACATACGGGTTATGATTGCC CTGCACCCACTTAACGGCTGACATGGG	Primers to replace *endA* gene with kan^R gene
539<br>540	GATCTGGCTGATTGCATACC<br>AAGCGCGTTGCACATACG	Primers to verify *endA::kan*^{R}
**List of the oligonucleotides used for qPCR**		
Q.bc1.F<br>Q.bc1.R	gatgtgcgctcattctcgta<br>acgcaccaaaaggtcacttc	qPCR the region 21.7 kb from *dif* before Chi sites on the left replichore
Q.bc3.F<br>Q.bc3.R	aaccactggaagacctggaa<br>gagcgaacggttttacttcg	qPCR the region 9.4 kb from *dif* before Chi sites on the right replichore
Q.Dif2.F<br>Q.Dif2.R	cgacattctaccgcctctga<br>ggcagtgcgtttcgttatgt	qPCR the region 30.3 kb from *dif* after Chi sites on the right replichore
Q.Dif1.F<br>Q.Dif1.R	cacgttaaatgaaaacccgcg<br>gaaggcgtcgaaaccaaaga	qPCR the region 27.3 kb from *dif* after Chi sites

		on the left replichore
